# Dithiocarbamate-inspired side chain stapling chemistry for peptide drug design†
†Electronic supplementary information (ESI) available. See DOI: 10.1039/c8sc03275k


**DOI:** 10.1039/c8sc03275k

**Published:** 2018-11-30

**Authors:** Xiang Li, W. David Tolbert, Hong-Gang Hu, Neelakshi Gohain, Yan Zou, Fan Niu, Wang-Xiao He, Weirong Yuan, Jia-Can Su, Marzena Pazgier, Wuyuan Lu

**Affiliations:** a School of Pharmacy , Second Military Medical University , Shanghai 200433 , China; b Institute of Human Virology and Department of Biochemistry and Molecular Biology , University of Maryland , School of Medicine , Baltimore , MD , USA . Email: mpazgier@ihv.umaryland.edu ; Email: wlu@ihv.umaryland.edu; c Changhai Hospital , Second Military Medical University , Shanghai 200433 , China . Email: drsujiacan@163.com

## Abstract

Two major pharmacological hurdles severely limit the widespread use of small peptides as therapeutics: poor proteolytic stability and membrane permeability. Importantly, low aqueous solubility also impedes the development of peptides for clinical use. Various elaborate side chain stapling chemistries have been developed for α-helical peptides to circumvent this problem, with considerable success in spite of inevitable limitations. Here we report a novel peptide stapling strategy based on the dithiocarbamate chemistry linking the side chains of residues Lys(*i*) and Cys(*i* + 4) of unprotected peptides and apply it to a series of dodecameric peptide antagonists of the p53-inhibitory oncogenic proteins MDM2 and MDMX. Crystallographic studies of peptide–MDM2/MDMX complexes structurally validated the chemoselectivity of the dithiocarbamate staple bridging Lys and Cys at (*i*, *i* + 4) positions. One dithiocarbamate-stapled PMI derivative, ^DTC^PMI, showed a 50-fold stronger binding to MDM2 and MDMX than its linear counterpart. Importantly, in contrast to PMI and its linear derivatives, the ^DTC^PMI peptide actively traversed the cell membrane and killed HCT116 tumor cells *in vitro* by activating the tumor suppressor protein p53. Compared with other known stapling techniques, our solution-based DTC stapling chemistry is simple, cost-effective, regio-specific and environmentally friendly, promising an important new tool for the development of peptide therapeutics with improved pharmacological properties including aqueous solubility, proteolytic stability and membrane permeability.

## Abstract 2

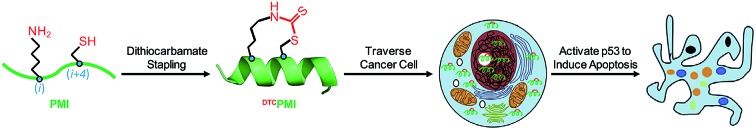
A novel peptide stapling strategy based on the dithiocarbamate chemistry linking the side chains of residues Lys(*i*) and Cys(*i* + 4) of unprotected peptides is developed.

## Introduction

Peptides are effective inhibitors of protein–protein interactions (PPI) and superior in many aspects as therapeutics to small molecule and protein drugs.^1^,^2^ However, peptides have two major pharmacological disadvantages – strong susceptibility to proteolytic degradation *in vivo* and poor membrane permeability,^3^–^5^ severely limiting their therapeutic efficacy. Importantly, another bottleneck in the development of peptides for clinical use is low solubility in aqueous solutions. Many therapeutic peptide drug candidates are abandoned because of their unacceptable solubility.^6^,^7^ For small peptides that adopt an α-helical structure upon interaction with target protein, various side chain stapling chemistries have been developed to improve their pharmacological properties *via* a pre-formed stable α-helix,^8^–^21^ among which the elaborate “hydrocarbon stapling” technique is probably best known.^22^–^24^ The hydrocarbon stapling chemistry takes advantage of Grubbs catalysts to crosslink on resin, *via* ruthenium-catalyzed olefin metathesis, two unnatural amino acids bearing olefinic side chains at (*i*, *i* + 4) or (*i*, *i* + 7) positions, and has been successfully used to design various peptide inhibitors with improved proteolytic stability, membrane permeability, and biological activity.^25^–^36^ One notable example is ALRN-6924, a hydrocarbon-stapled peptide antagonist of the oncogenic proteins MDM2 and MDMX that functionally inhibit the tumor suppressor protein p53.^37^–^39^ ALRN-6924, in phase 2 clinical trials for advanced solid tumors and lymphomas,^40^ kills tumor cells harboring wild-type p53 by antagonizing MDM2 and/or MDMX to reactivate the p53 pathway.

Despite its success in peptide drug design, hydrocarbon stapling can be technically cumbersome and costly due to the use of conformationally constrained unnatural amino acids and required transition metal carbene complexes as catalysts for olefin metathesis. Additionally, owing to an introduction of severely hydrophobic hydrocarbon stapling, another potential issue of this strategy is the problem of poor aqueous solubility, especially in those cases where the native hydrophilic side chains of Ser, Lys or Arg have to be sacrificed. To tackle these problems, we developed a novel peptide stapling strategy by crosslinking the side chains of Lys and Cys at (*i*, *i* + 4) positions *via* a thiocarbonyl group to form the dithiocarbamate (DTC) structure –NH–C(=S)–S–.

## Results and discussion

**Fig. 1:**
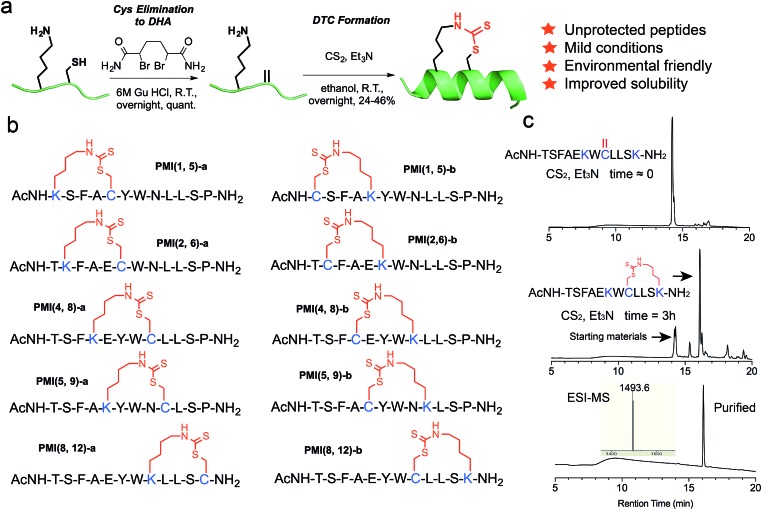
DTC stapling chemistry. (a) Schematic representation of the DTC chemistry linking the side chains of Lys and Cys at (*i*, *i* + 4) positions. (b) Structures of DTC-stapled PMI peptides. (c) Formation of the DTC staple as one predominant product from the PMI-derived peptide Ac-TSFAE**K**W**C**LLS**K**–NH_2_ according to HPLC analytic traces.

This solution chemistry for unprotected peptides entails the conversion of Cys *via* oxidative elimination to dehydroalanine (DHA),^41^,^42^ which subsequently reacts with the ε-amino group of Lys in the presence of carbon disulfide (CS_2_) (Fig. 1a).^43^–^45^ In this proof-of-concept study, we firstly used PMI – a potent dodecameric peptide antagonist of MDM2 and MDMX that, despite its high affinity for both proteins,^46^,^47^ fails to activate p53 and kill *p53*^+/+^ tumor cells due presumably to its inability to traverse the cell membrane and susceptibility to proteolytic degradation.^47^

Previous structural and functional studies of PMI (TS**F**AEY**W**NL**L**SP) identified Phe3, Trp7 and Leu10 as the most critical residues for MDM2/MDMX binding.^46^,^47^ Thus, we maintained those three residues in the design of DTC-stapled peptides and introduced Lys–Cys (a) or Cys–Lys (b) pairs into (1,5), (2,6), (4,8), (5,9), or (8,12) positions of PMI (Fig. 1b). These N-acetylated and C-amidated peptides were synthesized using solid phase peptide synthesis,^48^–^52^ and purified by HPLC to homogeneity. Conversion of Cys to DHA, monitored by HPLC and electrospray ionization mass spectrometry (ESI-MS), was achieved in an overnight reaction in 6 M GuHCl, pH 8.0, in the presence of the bisamide of the 1,4-dibromobutane core,^42^ to give the elimination-prone sulfonium salt, followed by HPLC purification. Crosslinking DHA and Lys side chains was readily accomplished overnight in ethanol containing Et_3_N and CS_2_ (Fig. 1a and S1†),^43^,^44^ as verified by ESI-MS (Fig. S2 and Table S1†), resulting in 10 DTC-stapled constructs termed PMI(1,5)-a, PMI(1,5)-b, PMI(2,6)-a, PMI(2,6)-b, PMI(4,8)-a, PMI(4,8)-b, PMI(5,9)-a, PMI(5,9)-b, PMI(8,12)-a and PMI(8,12)-b (Fig. 1b).

Although this work focused on PMI and its derivatives, the DTC stapling chemistry is expected to be applicable to other peptide systems as well. The transactivation domain (TAD) of p53, a peptide of 12–15 amino acid residues, has been extensively studied for its interaction with MDM2 and MDMX.^46^,^53^ We mutated Ser20 to Cys of a TAD peptide of p53, *i.e.*, ^16–27^p53 (QETF**S**DLW**K**LLP), and stapled it through a DTC linkage between Cys20 and Lys24 (Fig. S3†). Importantly, when Lys24 was replaced by ornithine, diaminobutyric acid or diaminopropionic acid, the DTC staple failed to form under otherwise identical experimental conditions, suggesting that the side chains of Cys and Lys (or Lys and Cys) at (*i*, *i* + 4) positions are optimally paired geometrically for the DTC chemistry.

To furthermore demonstrate the regio-selectivity of the DTC chemistry, we showed with the PMI-derived peptide Ac-TSFAE**K**W**C**LLS**K**–NH_2_, where Cys and two Lys residues are present in the same sequence. The question we asked was: can Cys form two competing DTC staples with the two Lys residues in the same sequence, at (*i*, *i* + 4) and (*i*, *i* + 2) positions? We recovered only one predominant reaction product containing a DTC staple (Fig. 1c), however. After HPLC purification, we subjected the product to tryptic digestion and mass spec analysis, and the data unambiguously demonstrated that the DTC staple had formed between Cys and Lys at (*i*, *i* + 4) positions, but not at (*i*, *i* + 2) positions (Fig. S4†).

It is worth noting that formation of the DTC crosslink between Lys and Cys side chains appears stereo-selective despite that Michael addition of Lys–NH–C(=S)S^–^ (product of the reaction between the amino group –NH_2_ and CS_2_) to dehydro-alanine could in theory yield two epimeric compounds (l-Cys and d-Cys) in equal quantities. In reality, however, one predominant isomer was identified and purified by HPLC for subsequent characterization (Fig. 1c and S2†), while a very minor isomer of an identical molecular mass was chromatographically resolved but discarded. To ascertain the purity of DTC-stapled peptides, we analyzed PMI(4,8)-a and PMI(8,12)-a on HPLC at different gradients. Both PMI(4,8)-a and PMI(8,12)-a, along with the wild type control peptide PMI-0, eluted as single and symmetric peaks at 30–60% and 35–45% acetonitrile over 30 min (Fig. S2†). While the stereo- and regio-selectivity of the DTC chemistry appears to be well-maintained in our study, a more rigorous examination of various reaction conditions and careful analysis of desired/undesired products is obviously warranted in the future to better understand the applicability of this stapling technique for peptide drug design.

**Fig. 2:**
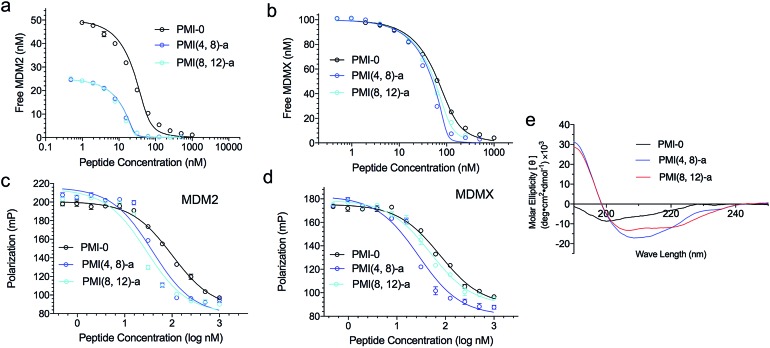
Characterization of representative DTC-stapled PMI peptides. (a) MDM2 at 25 or 50 nM and (b) MDMX at 100 nM with PMI-0, PMI(4,8)-a and PMI(8,12)-a as quantified by SPR-based competitive binding assays. (c) MDM2, (d) MDMX at 50 nM with PMI-0, PMI(4,8)-a and PMI(8,12)-a as quantified by FP-based competitive binding assays. *K*_d_ and *K*_i_ values were obtained through a non-linear regression analysis, and each curve is the mean of three independent measurements. Two replicates and three independent experiments were performed. (e) Circular dichroism spectra of PMI-0, PMI(4,8)-a and PMI(8,12)-a. The experiment was repeated independently twice with similar results.

We next evaluated the influence of DTC staple on binding affinities of peptides with target proteins. We quantified the interactions of DTC-stapled PMI peptides with the p53-binding domains of MDM2 and MDMX using fluorescence polarization (FP) and surface plasmon resonance (SPR) techniques as described,^46^,^47^,^55^–^57^ and the *K*_i_ and *K*_d_ values are tabulated in Table 1. In the FP-based competitive binding assay, stapled peptide at increasing concentrations competed off a fluorescently tagged PMI peptide (10 nM) complexed with synthetic ^25–109^MDM2/^24–108^MDMX (50 nM), resulting in a progressive decrease in FP. The equilibrium inhibition constant, *K*_i_, of stapled peptide for MDM2/MDMX was calculated as described.^54^ For SPR-based direct binding, different concentrations of stapled peptide were incubated with MDM2 at 50 nM or MDMX at 100 nM, unless indicated otherwise, and free MDM2/MDMX was quantified on a ^15–29^p53-immobilized CM5 sensor chip to obtain the equilibrium dissociation constant, *K*_d_, through non-linear regression analysis. Compared with the N-acetylated and C-amidated wild-type peptide PMI-0, PMI(4,8)-a and PMI(8,12)-a bound more strongly to MDM2 and MDMX. In fact, the crosslinked Lys–Cys pair at positions (4,8) enhanced peptide binding to both proteins by one order of magnitude as measured (Fig. 2a–d). Not surprisingly, both PMI(4,8)-a and PMI(8,12)-a partially adopted an α-helical structure in aqueous solution according to CD analyses (Table 1 and Fig. 2e), suggesting that crosslinking Lys–Cys side chains stabilized peptide conformation productive for MDM2 and MDMX binding. Similarly, the stapled p53 peptide bound to MDM2 and MDMX roughly one order of magnitude stronger than ^16–27^p53 (Table 1 and Fig. S3†). Of note, the reversal of Lys–Cys (a) to Cys–Lys (b) in PMI was in general detrimental to peptide binding to MDM2 and MDMX (Table 1), indicating that the DTC crosslink is functionally unidirectional.

**Table 1:** *K*_d_ and *K*_i_ values of DTC-stapled peptides for MDM2 and MDMX determined by SPR and FP techniques as well as percent α-helix measured by CD spectroscopy^*a*^

	PMI–MDM2		PMI–MDMX		α-Helix (%)
	*K*_i_ (nM)	*K*_d_ (nM)	*K*_i_ (nM)	*K*_d_ (nM)	
PMI-0	5.9 ± 2.6	4.2 ± 0.90	5.2 ± 1.0	17 ± 1.2	9.77
PMI(1,5)-a	123 ± 28	>500	>1000	>500	6.50
PMI(1,5)-b	51 ± 7.8	134 ± 5.5	39 ± 4.1	200 ± 7.1	8.38
PMI(2,6)-a	4.5 ± 1.8	6.2 ± 0.70	4.4 ± 1.2	9.7 ± 1.2	15.3
PMI(2,6)-b	337 ± 136	121 ± 5.5	17 ± 1.2	48 ± 3.4	4.81
PMI(4,8)-a	2.2 ± 4.0	0.35 ± 0.12	1.9 ± 2.5	0.82 ± 0.70	39.3
PMI(4,8)-b	14 ± 1.5	20 ± 1.9	5.7 ± 1.7	12 ± 1.8	9.15
PMI(5,9)-a	29 ± 3.4	55 ± 3.3	24 ± 3.2	89 ± 5.6	6.88
PMI(5,9)-b	69 ± 13	90 ± 4.6	20 ± 2.7	54 ± 3.4	9.58
PMI(8,12)-a	1.7 ± 3.7	0.18 ± 0.19	3.3 ± 1.3	6.0 ± 0.90	43.3
PMI(8,12)-b	38 ± 6.5	57 ± 3.0	162 ± 31	400 ± 16	0.23
^DTC^PMI Ctrl.	42 ± 4.0	47 ± 3.0	47 ± 3.1	220 ± 11	16.6
^DTC^PMI	2.1 ± 2.7	0.87 ± 0.49	2.0 ± 1.5	3.9 ± 2.0	62.2
p53	>1000	346 ± 19	987 ± 17	614 ± 26	N/A
^DTC^p53	16 ± 1.2	46 ± 2.7	12 ± 1.3	62 ± 4.9	N/A

^*a*^In the SPR-based quantification method, where direct binding of stapled peptide to MDM2/MDMX was measured, *K*_d_ (the equilibrium dissociation constant) values were given by a non-linear regression analysis using the equation *K*_d_ = [peptide][MDM2/MDMX]/[complex]. In the FP-based competitive binding assay, where a fluorescently tagged PMI peptide in complex with MDM2/MDMX was competed off by stapled peptide, *K*_i_ (equilibrium inhibition constant) values were calculated using the equation *K*_i_ = [I]_50_/([L]_50_/*K*_d_ + [P]_0_/*K*_d_ + 1),^54^ in which [I]_50_ denotes the concentration of stapled peptide at 50% inhibition, [L]_50_ the concentration of labeled PMI at 50% inhibition, [P]_0_ the concentration of free MDM2/MDMX at 0% inhibition, and *K*_d_ the equilibrium dissociation constant of the MDM2/MDMX–PMI complex.

**Fig. 3:**
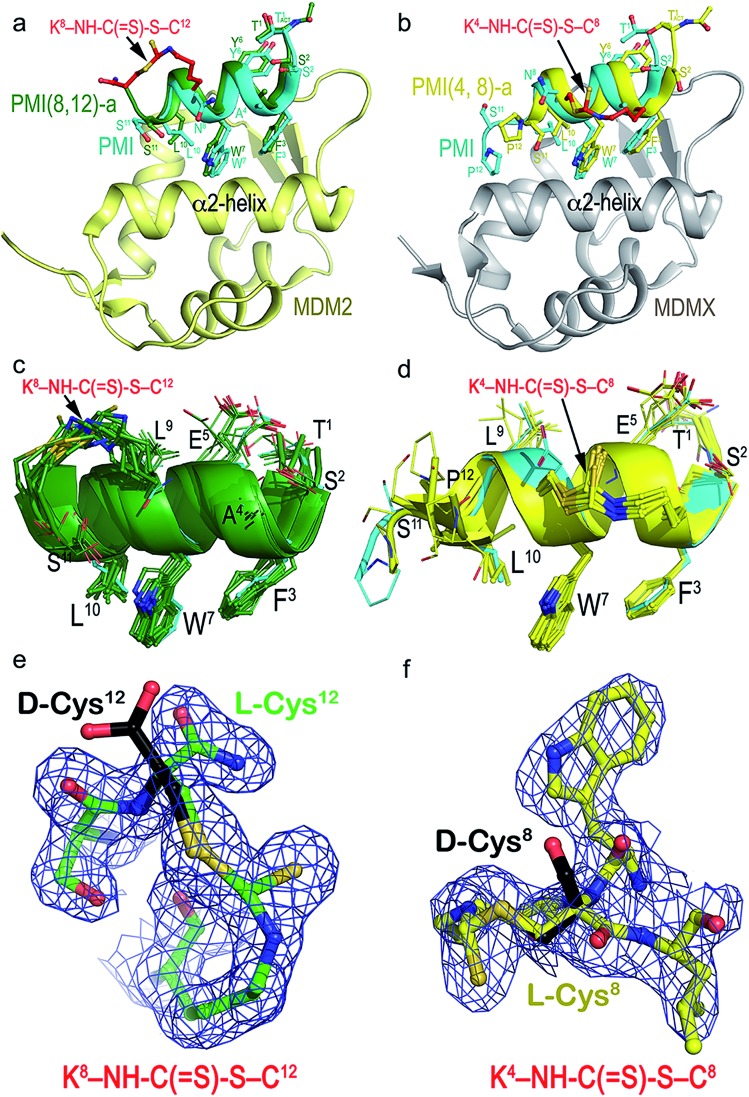
Structural validation of DTC staples. (a) Co-crystal structure of PMI(8,12)-a (green) or PMI (cyan) in complex with MDM2 (yellow). (b) Co-crystal structure of PMI(4,8)-a (yellow) or PMI (cyan) in complex with MDMX (gray). (c and d) Superposition of PMI(8,12)-a and PMI(4,8)-a peptides from the crystal asymmetric unit to each other and to the parent PMI peptide. PMI(8,12)-a and PMI(4,8)-a peptides could be superimposed with an average RMSD value of 0.946 Å and 0.943 Å for the main chain atoms of 11 residues (Thr1–Ser11) among themselves and with PMI peptides, respectively. (e and f) The electron density maps of the DTC staples seen in PMI(8,12)-a (left) and PMI(4,8)-a (right) contoured at 1.0*σ* level. d-Cysteine in black is modeled at the same position, where no electron density was observed.

To structurally validate the DTC stapling chemistry, we solved the co-crystal structures of MDM2–PMI(8,12)-a and MDMX–PMI(4,8)-a at 1.8 and 2.7 Å resolution (Table S2†), respectively, and compared them with the structures of MDM2 and MDMX in complex with PMI (Fig. 3a and b).^47^ Both complexes crystallized with multiple copies in the asymmetric unit of the crystal – 12 for MDM2–PMI(8,12)-a and 8 for MDMX–PMI(4,8)-a (Table S2 and Fig. S6†). Whereas all 12 residues could be built into each PMI(8,12)-a peptide complexed with MDM2, PMI(4,8)-a was fully defined in only 3 copies of the MDMX complex with no density observed for Ser11 and/or Pro12 (Fig. 3c and d). Alignment analysis of the PMI(8,12)-a conformation also indicated noticeable variability among the 12 copies of peptide, as evidenced by the root-mean-square deviation (RMSD) between the main-chain atoms in the range of 0.48–1.35 Å (Table S3†). In both complexes, however, the crystallographic density for all atoms of the crosslink formed between Lys(*i*) and Cys(*i* + 4) unambiguously defined the geometry of the DTC staple.

As shown in Fig. 3a, MDM2-bound PMI(8,12)-a largely overlapped with PMI, differing mainly in positions of the equivalent C_α_ atoms of residues Thr1–Trp7 with little change in the C-terminal region (Trp7–Ser11) (Table S3†). More pronounced differences were observed between MDMX-bound PMI(4,8)-a and PMI (Table S4†), with the backbone of the former longitudinally shifting ∼2 Å toward one side of the p53-binding pocket of MDMX and closer to its α2-helix in relation to PMI (Fig. 3b). This shift, while increasing PMI(4,8)-a contacts with the edge of the cavity formed by the α2-helix of MDMX, reduced hydrophobic contacts and lengthened some hydrogen bonds seen in the PMI–MDMX complex (Fig. S7†). The DTC staple rigidified, at positions (8,12), the C-terminus of PMI in a helical conformation and extended, at positions (4,8), the C-terminal helix of PMI from Leu9 to Ser11 (Fig. 3a and b). The rigidity of PMI(8,12)-a or PMI(4,8)-a increased to such an extent that the local buried surface area (BSA) slightly decreased as compared with the BSA contributed by PMI to its interface with MDM2/MDMX (Fig. S8†). This finding suggests that DTC stapling-enhanced binding may be energetically attributable to a reduced loss in entropy afforded by a pre-organized stable helix.

We deduced the DTC structure of the predominant epimer from the crystal structures of PMI(4,8)-a and PMI(8,12)-a in respective complex with MDMX and MDM2, where Cys8 or Cys12 remained as an l-amino acid residue as shown in the electron density maps (Fig. 3e and f). Our biochemical and biophysical findings on the DTC-stapled peptides unambiguously demonstrated their purity and stereo-selectivity for l-Cys, though.

Side chain stapled peptides are structurally rigidified as compared with their linear counterparts and, thus, expected to be more resistant to proteolysis *in vivo*. We used HPLC and ESI-MS to evaluate the proteolytic stability of PMI(8,12)-a *versus* PMI-0 at 100 μM in cell culture medium in the presence of 25 μg ml^–1^ cathepsin G – an intracellular protease with dual specificities for both basic and bulky hydrophobic residues.^58^ As shown in Fig. S9,† while PMI-0 was fully degraded by the enzyme within 30 min of co-incubation at room temperature, the DTC-stapled peptide was substantially more stable with a half-life of ∼8 h under identical conditions. Similar results were obtained using human serum (Fig. S9†). Of note, the DTC structure is also stable in the presence of reduced glutathione (GST). When PMI(8,12)-a was incubated at 25 °C in PBS buffer with GST at 10 mM – a physiological concentration,^59^ no apparent breakdown of the DTC structure was observed over 24 h (Fig. S9†).

**Fig. 4:**
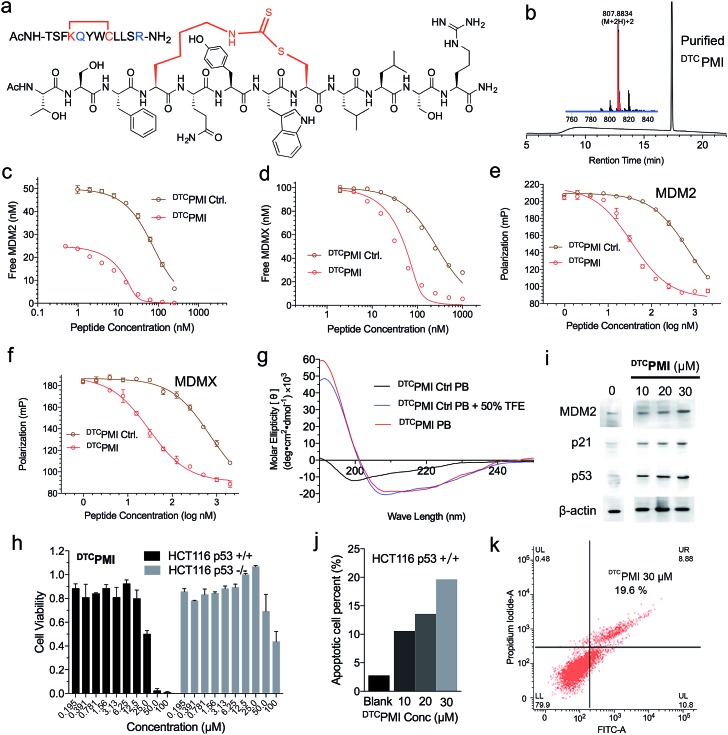
Design and functional characterization of ^DTC^PMI. (a) Amino acid sequence and chemical structure of ^DTC^PMI. (b) HPLC chromatograms and MS spectra of ^DTC^PMI. (c) MDM2 at 25 or 50 nM and (d) MDMX at 100 nM with ^DTC^PMI Ctrl. and ^DTC^PMI as quantified by SPR-based competitive binding assays. (e) MDM2, (f) MDMX at 50 nM with ^DTC^PMI Ctrl. and ^DTC^PMI as quantified by FP-based competitive binding assays. *K*_d_ and *K*_i_ values were obtained through a non-linear regression analysis, and each curve is the mean of three independent measurements. Two replicates and three independent experiments were performed. (g) Circular dichroism spectra of ^DTC^PMI. (h) Dose-dependent anti-proliferative activity of ^DTC^PMI against isogenic HCT116 *p53*^+/+^ and *p53*^–/–^ cell lines. (i) Western blot analysis of the expression of *MDM2*, *p21* and *p53* in HCT116 *p53*^+/+^ cells treated with ^DTC^PMI. (j and k) ^DTC^PMI-induced apoptosis of HCT116 *p53*^+/+^ cells as analyzed by flow cytometry. The experiment was repeated independently twice with similar results.

Verdine and colleagues have shown that structurally permissible stapling of a p53 peptide, while enhancing α-helicity and improving MDM2 binding, is not sufficient to endow the peptide with an ability to kill tumor cells.^22^ Although cationicity is not a universal molecular signature of cell-penetrating peptides, it plays a critical role in the ability of stapled peptides to traverse the cell membrane to exert biological activity.^10^,^22^,^28^ Perhaps not surprisingly, our DTC-stapled peptides carrying a net charge of either 0 or –1 showed little cytotoxicity against HCT116 *p53*^+/+^ and HCT116 *p53*^–/–^ cells at up to 100 μM (Fig. S10†). Using PMI(4,8)-a as a template, we made two cationicity-enhancing mutations, E5Q and P12R, resulting in a DTC-stapled peptide termed ^DTC^PMI with a +1 net charge (Fig. 4a and b). Confocal microscopic analysis of HCT116 cells treated with 20 μM ^DTC^PMI N-terminally conjugated to fluorescein (FITC) revealed a diffused intracellular localization of the peptide (Fig. S11†), confirming the ability of ^DTC^PMI to permeabilize the cell membrane.

Compared with its unstapled control peptide, Ac-TSF**K**QYW**C**LLSR–NH_2_, DTC crosslinking increased peptide binding affinity for MDM2 and MDMX by 50-fold as measured by SPR (Fig. 4c, d and Table 1) or ∼20-fold by FP (Fig. 4e, f and Table 1), making ^DTC^PMI (*K*_d_ = 0.87 and 3.9 nM for MDM2 and MDMX, respectively) a strong dual-specificity peptide antagonist against both proteins.^46^,^47^,^60^ Of note, ^DTC^PMI also displayed a strong tendency to adopt α-helix on its own in aqueous solution (Table 1 and Fig. 4g), likely contributing energetically to its high-affinity binding to both MDM2 and MDMX. As is the case with ^DTC^PMI, PMI(4,8)-a and PMI(8,12)-a, while stapling-enhanced α-helicity qualitatively predicts strong peptide binding to MDM2/MDMX, a quantitative correlation appears lacking, due, in part, to the deficiency of CD spectroscopy in accurate measurements of α-helicity of small peptides that are generally disordered and conformationally heterogeneous.

To functionally validate ^DTC^PMI, we subjected it and its unstapled control to a cell viability assay using HCT116 *p53*^+/+^ and *p53*^–/–^ cells. Lane and colleagues previously reported that serum proteins were inhibitory against the tumor-killing activity of hydrocarbon-stapled peptide antagonists of MDM2.^27^ To mitigate the potential effect of serum binding on peptide activity, we treated cells in serum-free media for 8 h, followed by addition of serum supplements and incubation for 64 h. While the control peptide exhibited no anti-proliferative activity against both cell lines at concentrations of up to 50 μM (Fig. S12†), ^DTC^PMI displayed p53-dependent growth inhibitory activity against HCT116 *p53*^+/+^, but not HCT116 *p53*^–/–^, with an IC_50_ value of ∼25 μM at 72 h (Fig. 4h and S13†). To investigate the mechanisms of killing of HCT116 *p53*^+/+^ by ^DTC^PMI, we analyzed the expression of *MDM2*, *p53* and *p21* by western blotting. As shown in Fig. 4i and S14,† 8 h after treatment with ^DTC^PMI, dose-dependent induction of p53, MDM2 and p21 became evident in HCT116 *p53*^+/+^ cells. Consistent with this result, dose-dependent induction of apoptosis of HCT116 *p53*^+/+^ cells by ^DTC^PMI was verified by fluorescence-activated cell sorting (FACS) (Fig. 4j, k and S15†). By contrast, no obvious apoptosis of HCT116 *p53*^–/–^ cells was observed by FACS under identical treatment conditions (Fig. S16†). Taken together, these findings support that ^DTC^PMI actively traversed the cell membrane and killed tumor cells by antagonizing MDM2 to reactivate the p53 pathway. It is worth pointing out that as is often the case with other stapled peptide activators of p53,^22^,^26^ ^DTC^PMI, despite its low nano-molar binding affinity for MDM2 and MDMX, is rather weak in killing HCT116 *p53*^+/+^ cells. The weak *in vitro* activity implies that stapling alone is insufficient to achieve optimal therapeutic efficacy of helical peptides, dictated by cell internalization, endosomal escape, proteolytic stability, spatio-temporal distribution, *etc.*

Of note, at the high concentration of 100 μM, ^DTC^PMI significantly reduced cell viability of HCT116 *p53*^–/–^ cells as well (Fig. 4h). This finding is not entirely surprising in light of the fact that the MDM2 antagonist Nutlin-3 also kills HCT116 *p53*^–/–^ at high concentrations, in part by disrupting MDM2 interactions with p73,^61^ a member of the p53 family that transcriptionally induces cell-cycle arrest and/or apoptosis.^62^ In fact, recent data demonstrate that p73 is elevated to compensate for p53 loss when *MDM2* is deleted in p53-null tumor cells.^63^ It is therefore plausible that the observed killing of HCT116 *p53*^–/–^ by ^DTC^PMI at high concentrations arises from its p53-independent on-target activity, potentially extending ^DTC^PMI to the treatment of p53-deficient cancers as well.

Aside from the simplicity of using natural amino acids, the DTC chemistry may offer an added advantage over the hydrocarbon stapling technique: peptide solubility. If stapling severely decreases peptide solubility, it can potentially limit drug concentration *in vivo*, thus therapeutic efficacy. For direct comparison, we stapled Ac-TSF**X**QYW**X**LLSR–NH_2_ with a hydrocarbon linkage between **X** residues at positions 4 and 8 (**X** = (*S*)-2-(4′-pentenyl)alanine), yielding a hydrocarbon stapled peptide termed ^HC^PMI that differs only in the crosslink from ^DTC^PMI. ^DTC^PMI and ^HC^PMI were each suspended at 20 mg ml^–1^ in PBS, followed by a 2-fold serial dilution and OD measurements at 600 nm. As shown in Fig. S17,† while ^DTC^PMI was soluble at a concentration of >10 mg ml^–1^, the solubility of ^HC^PMI was significantly lower, at ∼0.3 mg ml^–1^. Since dithiocarbamate contains multiple hydrogen bond donors/acceptors, the DTC staple is expected to be more soluble than all-hydrocarbon crosslinks.

## Conclusions

We have developed a novel stapling strategy for peptide drug design by taking advantage of the DTC chemistry to crosslink the side chains of the two natural amino acid residues Lys and Cys at (*i*, *i* + 4) positions. The DTC staple, structurally validated, induced the formation of and stabilized a productive α-helical conformation of PMI – a dual-specificity peptide antagonist of MDM2 and MDMX, enabling it to traverse the cell membrane and kill tumor cells by reactivating the p53 pathway. DTC stapling functionally rescued PMI that, on its own, failed to activate p53 *in vitro* and *in vivo* due to its poor membrane permeability and susceptibility to proteolytic degradation. It is worth noting that DTC stapling offers a better peptide aqueous solubility over hydrocarbon stapling. Compared with other known stapling techniques, the solution-based DTC chemistry is simple, cost-effective, regio-specific, and environmentally friendly, promising an important new tool for peptide drug discovery and development for a variety of human diseases.

## Author contributions

XL, HGH, MP and WL conceived and designed the study. XL, WDT, NG, YZ, FN, WH and WY performed the experiments. HGH and JCS helped with study design, and edited the manuscript. XL, MP and WL wrote the paper. All authors read and approved the manuscript.

## Conflicts of interest

There are no conflicts to declare.

## Supplementary Material

Supplementary information
