# Supplementary material for: Dithiocarbamate-inspired side chain stapling chemistry for peptide drug design
Source: Chem Sci. 2018 Nov 30;10(5):1522–30. doi: 10.1039/c8sc03275k (PMC6357863; doi:10.1039/c8sc03275k)
Supplement: Supplementary file 1 [file SC-010-C8SC03275K-s001.pdf]

Electronic Supplementary Material (ESI) for Chemical Science.
This journal is © The Royal Society of Chemistry 2018

Supporting information

Dithiocarbamate-inspired Side Chain Stapling Chemistry for Peptide Drug Design

Xiang Li,[+a,b] W. David Tolbert,[+b] Hong-Gang Hu,[+a] Neelakshi Gohain,[b] Yan Zou,[a] Fan Niu,[b] Wang-Xiao He,[b] Weirong Yuan,[b] Jia-Can Su,[*c] Marzena Pazgier,[*b] and Wuyuan Lu[*b]

a. School of Pharmacy, or c. Changhai Hospital, Second Military Medical University, Shanghai 200433, China. Email: drsujiacan@163.com

b. Institute of Human Virology and Department of Biochemistry and Molecular Biology, University of Maryland School of Medicine, Baltimore, MD, USA

Email: wlu@ihv.umaryland.edu; mpazgier@ihv.umaryland.edu

Table of Contents

Materials. 4

Reversed phase analytical HPLC. 4

High resolution mass spectra. 4

Peptide and protein synthesis. 4

Synthesis of Stapled PMIs. 4

Surface plasmon resonance (SPR) 4

Fluorescence polarization (FP). 5

Cell Viability Assay. 5

Circular Dichroism (CD) Spectroscopy. 5

Proteolytic Stability. 5

Stability in GSH. 5

Cellular uptake of ^{DTC}PMI. 5

Western Blot Analysis. 6

Cell Apoptosis Assay. 6

All Hydrocarbon Stapling. 6

Comparison of Solubility between DTC and Hydrocarbon link. 6

Crystallization of stapled-PMI complexes. 7

Data Collection, Structure Solution and Refinement. 7

Fig. S1 The synthetic route of DTC-stapled PMI(1,5)-a 8

Fig. S2 HPLC and MS chromatograms of DTC-stapled peptides. 9

Fig. S3 HPLC and MS chromatograms of ^{DTC}p53 and corresponding binding curves with MDM2 (left) and MDMX (right) as determined by SPR and FP. 10

Fig. S4 Characterization of the DTC staple from the PMI-derived peptide Ac-TSFAEKWCLLSK-NH_2. 11

Fig. S5 Circular dichroism spectra of DTC-stapled PMIs 12

Fig. S6 Superposition of MDM2-PMI(8,12)-a and MDMX-PMI(4,8)-a copies within the asymmetric unit of each crystal form.. 13

Fig. S7 MDM2-PMI(8,12)-a and MDMX-PMI(4,8)-a complex interfaces 14

Fig. S8 Structural analysis of interactions of stapled PMI with MDM2 and MDMX. 15

Fig. S9 Stability of PMI-0 and/or PMI(8,12)-a in the presence of cathepsin G or GSH or human serum. 16

Fig. S10 Viability of HCT116 $p53^{+/+}$ and HCT116 $p53^{-/-}$ cell lines in the presence of PMI-0 and stapled PMIs. ... 17

Fig. S11 Confocal microscope images of FITC-labeled ^{DTC}PMI Ctrl. and ^{DTC}PMI localization in HCT116 cells. 18

Fig. S12 Viability of HCT116 $p53^{+/+}$ and $p53^{-/-}$ cell lines in the presence of linear ^{DTC}PMI control 19

Fig. S13 Fitted curves of the viability of HCT116 *p53*$^{+/+}$ and $p53^{-/-}$ cell lines in the presence of ^{DTC}PMI 20

Fig. S14 Quantitative western blot analysis treated with different concentrations of ^{DTC}PMI. 21

Fig. S15 ^{DTC}PMI-induced apoptosis of HCT 116 $p53^{+/+}$ cells as measured by flow cytometry. 22

Fig. S16 ^{DTC}PMI-induced apoptosis of HCT 116 $p53^{-/-}$ cells as measured by flow cytometry. 23

Fig. S17 Comparison of Solubility between DTC and Hydrocarbon link. 24

Table S1 Yield and HR-MS spectrometry data for DTC-stapled PMI peptides. 25

Table S2 Data collection and refinement statistics 26

Table S3 The root mean square deviation (RMSD) between MDM2-PMI(8,12)-a and MDM2-PMI complexes..27

Table S4 The root mean square deviation (RMSD) between MDMX-PMI(4,8)-a and MDMX-PMI complexes..30

References 31

Materials. All reagents and solvents were purchased from Peptide International, Bachem Co. Ltd, Sigma or Millipore, and were purified when necessary.

Reversed phase analytical HPLC. Analytical HPLC was run on a SHIMADZU (Prominence LC-20AD) instrument using an analytical column (Dikma Tech "Diamonsil Plus C18", 250 × 4.6 mM, 5 μm particle size, flow rate 1.0 mL/min, r.t.). Analytical injections were monitored at 214 nm. Solution A was 0.1% TFA in water, and solution B was 0.1% TFA in MeCN. Gradient A: A linear gradient of 10% to 10% B over 2 mins, then a linear gradient of 10% to 80% B over 25 mins.

High resolution mass spectra. HR-Q-TOF-MS was measured on an Agilent 6538 UHD Accurate Mass Q-TOF mass spectrometer.

Peptide and protein synthesis. All peptides and proteins used in this work were chemically synthesized, either in a stepwise fashion or via native chemical ligation.[1-3] Peptides were synthesized using a machine-assisted Boc chemistry tailored from the optimized HBTU activation/DIEA in situ neutralization protocol.[4] After chain assembly, side chain protecting groups were removed and peptides cleaved from the resin by treatment with anhydrous HF and p-cresol (9:1) at 0 °C for 1 h. Crude peptides were precipitated with cold ether and purified by preparative C18 reversed-phase (RP) HPLC. The synthesis of $^{25\text{-}109}$MDM2 and $^{24\text{-}108}$MDMX was described previously,[3] and obtained via native chemical ligation. The reaction between MDM2(25-76)-COSR and MDM2(77–109) (1.5 eq) or between MDMX (24–75)-COSR and MDMX (76–108) (1.5 eq) was carried out at a total peptide concentration of 10-20 mg/ml in 0.25 M phosphate buffer (pH 7.1) containing 6 M guanidine hydrochloride, 50 mM MPAA and 20 mM TCEP•HCl. They went to completion overnight as monitored by analytical HPLC. The ligation products were purified by preparative RP-HPLC to homogeneity. The molecular masses of all peptides and proteins were ascertained by electrospray ionization mass spectrometry.

Synthesis of Stapled PMIs. PMI(1,5)-a is used as an example (**Fig. S17**). **Cys to Dha.** Buffer A containing 6 M guanidine hydrochloride and 100 mM Na_2HPO_4, pH = 8.5, and Buffer B containing 6 M guanidine hydrochloride and 100 mM NaH_2PO_4, pH = 2.5, were prepared prior to the reaction. 3 mL Buffer B was used for dissolving 50 mg PMI(1K,5C) for storage. 75 mg Bisamide reagent (1.5 mg per linear peptide) was dissolved in 47 mL Buffer A, followed by a slow addition of Buffer B containing the linear peptide. The reaction was stirred at room temperature overnight and monitored by analytical HPLC. The crude intermediate product PMI(1K,5DHA) was purified by preparative RP-HPLC to homogeneity (35 mg). **DTC cyclization**. 20 mg PMI(1K,5DHA) was dissolved in 10 mL ethanol, followed by addition of 1mL Et_3N and 1mL CS_2. The reaction proceeded with stirring overnight at room temperature until a complete conversion. After the solvent was removed, the residual material was purified by preparative RP-HPLC to yield the stapled product PMI(1,5)-a (10 mg).

Surface plasmon resonance (SPR). Competition binding kinetics was carried out at 25 °C using a Biacore T100 SPR instrument and $^{15\text{-}29}$p53-immobilized CM5 sensor chips as described.[3,5-10] $^{25\text{-}109}$MDM2 and $^{24\text{-}108}$MDMX at 50 nM or 100 nM were incubated in 10 mM HEPES buffer containing 150 mM NaCl, 0.005% surfactant P20, pH 7.4, with varying concentrations of peptide inhibitor before SPR analysis. The concentration of unbound

MDM2 or MDMX in solution was deduced, based on p53-association RU values, from a calibration curve established by RU measurements of different concentrations of MDM2/MDMX injected alone. Two replicates and three independent experiments were performed.

Fluorescence polarization (FP). A FP-based competitive binding assay was established using $^{25\text{-}109}$MDM2, $^{24\text{-}108}$MDMX and a fluorescently tagged PMI peptide as previously described.[5,9-10] Succinimidyl ester-activated carboxytetramethylrhodamine (TAMRA-NHS) was covalently conjugated to the N-terminus of PMI (TSFAEYWNLLSP) ($K_d^{PMI–MDM2}$ = 3.2 nM, $K_d^{PMI–MDMX}$ = 8.5 nM). Unlabeled PMI competed with TAMRA-PMI for MDM2/MDMX binding, based on which the K_d values of TAMRA-PMI with MDM2 and MDMX were determined by changes in FP to be 0.62 and 0.72 nM, respectively. As an additional positive control, we quantified the binding of Nutlin-3 to MDM2 and MDMX, yielding respective Ki values of 5.1 nM and 1.54 μM, similar to the values reported in the literature.[11] For dose-dependent competitive binding experiments, MDM2 or MDMX protein (50 nM) was first incubated with TAMRA-PMI peptide (10 nM) in PBS (pH 7.4) on a Costar 96-well plate, to which a serially diluted solution of test peptide was added to a final volume of 125 μL. After 30 min of incubation at room temperature, the FP values were measured at λ_{ex}=530 nm and λ_{em}=580 nm on a Tecan Infinite M1000 plate reader. Curve fitting was performed using GRAPHPAD PRISM software, and Ki values were calculated as described previously.[5,9-10] Two replicates and three independent experiments were performed.

Cell Viability Assay. The human colon cancer cell lines HCT116 p53$^{+/+}$ and HCT116 p53$^{-/-}$ were generously provided by Prof. Vogelstein of Johns Hopkins University, and maintained in McCoy's 5A medium (Invitrogen) supplemented with 10% heat-inactivated FCS and 1% penicillin-streptomycin at 37 °C with 5% CO_2 under fully humidified conditions. Cells (3×10^3 cells/well) were seeded at in 96-well plates and treated with PMI and stapled PMIs at various concentrations in serum-free media for 8 hours, followed by serum complementary and additional incubation for 64 hours. The absorbance at 450 nm was then measured followed by the addition of CCK8 kit, and percent cell viability was calculated on the ratio of the A_{450} of sample wells versus reference wells.

Circular Dichroism (CD) Spectroscopy. Compounds were dissolved in PB (pH=7.2) to concentrations ranging from 10-50 μM. The spectra were obtained on a Jasco J-715 spectropolarimeter at 20 °C. The spectra were collected using a 0.1 cm path-length quartz cuvette with the following measurement parameters: wavelength, 185-255 nm; step resolution 0.1 nm; speed, 20 nm min^{-1}; accumulations, 6; bandwidth, 1 nm. The helical content of each peptide was calculated as reported previously.[12]

Proteolytic Stability. PMI-0 and the stapled peptide PMI(8,12)-a were incubated at 100 μM each in RPMI 1640 with 25 μg/ml cathepsin G – an intracellular protease with dual specificities for both basic and bulky hydrophobic residues. RP-HPLC was used to monitor and quantify time-dependent peptide hydrolysis.

Stability in GSH. PMI(8,12)-a was incubated at 25 °C in PBS buffer with reduced glutathione at 10 mM. RP-HPLC and ESI-MS were used to monitor and quantify time-dependent breakdown of the DTC staple.

Cellular uptake of ^{DTC}PMI. A fluorescent FITC moiety was appended via an aminocaproic acid to the N-terminal of ^{DTC}PMI. HCT116 p53$^{+/+}$ cells were seeded in four-well chambered cover-glass (6×10^4 cells per well) and allowed to grow overnight. Cells were then incubated with 20 μM FITC-^{DTC}PMI for 4 h. Cells were washed with Dulbecco's phosphate buffered saline, fixed with 4% (wt/vol) paraformaldehyde, finally incubated by DAPI to stain the cell nucleus. Imaged using an LSM 510 Zeiss Axiovert 200M (v4.0) confocal microscope. Images were analyzed using an LSM image browser.

Western Blot Analysis. HCT116 p53$^{+/+}$ cells (1×10^6) incubated at 37 °C were treated with ^{DTC}PMI (10, 20, 30 μM) in serum-free media for 8 hours. The cells were lysed (20 mM Tris-HCl pH 8.0, 0.8 % SDS, 1 mM PMSF, 1 U mL^{-1} benzonase nuclease) and the crude lysates were clarified by brief centrifugation and total protein concentration was determined by using the Pierce BCA protein assay. Aliquots containing 5 μg of total protein were run on 4-12% Bis-Tris polyacrylamide gels (Invitrogen). Proteins were detected by chemiluminescence reagent (Perkin Elmer) using antibodies specific for p53 (Santa Cruz Biotechnology), MDM2 (Santa Cruz Biotechnology), p21 (Merck Millipore), and β-actin (Sigma-Aldrich).

Cell Apoptosis Assay. HCT116 p53$^{+/+}$ or HCT116 p53$^{-/-}$ cells were seeded in 6-well tissue culture plates (3×10^5 cells per well) for 12 h and treated with 10, 20, or 30 μM ^{DTC}PMI in serum-free media for 8 hours, followed by serum complementary and additional incubation for 40 hours. No treatment controls were established. Culture medium that may contain detached cells was collected, and attached cells were trypsinized. After centrifugation and removal of the supernatants, cells were resuspended in 300 μL of 1×binding buffer which was then added to 5 μL of annexin V-FITC and incubated at room temperature for 15 min. After addition of 10 μL of PI, the cells were incubated at room temperature for another 10 min in the dark. The stained cells were analyzed by a flow cytometer (BD-FACSVerse).

All Hydrocarbon Stapling. 400 mg Rink Amide MBHA resin was swelled with DCM (5 mL) for 20 mins. Then the resin was treated with 20% piperidine/DMF twice (10 and 5 mins), followed by washing with DMF (5 times), DCM (5 times) and DMF (5 times). For coupling of the first amino acid, Fmoc-AA-OH (1 mmol), HCTU (0.9 mmol), DIEA (2 mmol) and DMF (6 mL) were mixed for 2 mins and then added to the resin. After 2 hrs, the resin was washed with DMF (5 times), DCM (5 times), and DMF (5 times). The peptide couplings of N-Fmoc-*α*-pentene amino acid S_5 were carried out over a single two hours coupling cycle using 2 eq. of the Fmoc protected amino acids. The deprotection, washing, coupling and washing steps were repeated until all the amino acid residues were assembled reagent. The peptide-bound resin was treated with 20% piperidine/DMF to remove the Fmoc group from the N-terminus. After the resin was washed it was treated with 3 mL solution of acetic anhydride and pyridine (1:1) for 20 mins. Then the resin was washed with DMF (5 times), DCM (5 times), and DMF (5 times). The ring-closing metathesis reaction was carried out in 1,2-dichloroethane (DCE) at room temperature (20-25 °C) using Grubbs' first-generation catalyst (10 mM). After the first round of the 2 hrs metathesis, we repeated the same procedure for a second round of catalyst treatment with fresh catalyst solution, then the peptide-resin was washed with DMF (5 times), DCM (5 times). Peptides were cleaved from their resin

by treatment with reagent K (80% TFA, 5%, H_2O, 2.5% EDT, 5% Thioanisole and 7.5% phenol) for 4 hrs at room temperature. After completion of the cleavage reaction, TFA was evaporated by blowing with Ar. The crude peptides were obtained by precipitation with 40 mL of cold diethyl ether and purified with preparative RP-HPLC to yield the stapled product (^{HC}PMI).

Comparison of Solubility between DTC and Hydrocarbon link. Mix 1 mg ^{DTC}PMI and ^{HC}PMI in 50 μL PBS Buffer individually, and then transfer 40 μL the suspension to the Costar mini 96-well plate. The suspension was gradient diluted from 20 mg/mL to 0.0195 mg/mL. OD values were measured at 600 nm on a Biotech Synergy 4 plate reader. PBS was set as blank control.

Crystallization of stapled-PMI complexes. Initial screening for crystals was done with an Art Robbinson crystallization robot using vapor diffusion sitting trials of sparse matrix crystallization screens: the Hampton crystal screen I and II (Hampton Research), the precipitant wizard screen (Emerald BioSystems), the synergy screen (Emerald BioSystems) and the ProComplex and MacroSol screens from Molecular Dimensions. All crystallization experiments were performed with complexes at 8-10 mg/ml in 20 mM Tris pH 7.4. Conditions that produced micro crystals were then reproduced and optimized using the hanging-drop vapor diffusion method (drops of 0.5 μl of protein and 0.5 μl of precipitant solution equilibrated against 700 μl of reservoir solution). Diffraction quality crystals for MDM2-PMI(8,12)-a complex were obtained from a solution containing 1.34 M ammonium sulfate, 6.7% (v/v) glycerol, 50 mM magnesium sulfate, and 0.1 M imidazole pH 6.5. Prior to being frozen, the crystals were transferred into the crystal growth solution supplemented with 20% (v/v) 2-methyl-2,4-pentanediol (MPD). Crystals of MDMX-PMI(4,8)-a complex were grown from 30% (v/v) 2-propanol, 30% (v/v) PEG 3350, and 0.1 M Tris-HCl pH 8.5 and frozen from the same solution supplemented with 20% (v/v) MPD.

Data Collection, Structure Solution and Refinement. Diffraction data for both complexes were collected at the Stanford Synchrotron Radiation Light Source (SSRL) BL12-2 beam line equipped with Pilatus 6M PAD area detector. The MDM2-PMI(8,12)-a complex crystals belong to a space group $C222_1$ with unit-cell parameters a = 90.8 Å, b = 157.5 Å, and c = 196.7 Å with twelve complexes copies present in the asymmetric unit. The MDMX-PMI(4,8)-a complex crystals belong to a space group P1 with unit-cell parameters a = 43.3 Å, b = 47.7 Å, c = 93.4 Å, α = 76.7º, β = 89.9º, and γ = 72.6º and eight complexes in the asymmetric unit (**Table S3**). The data for both the complexes were processed and scaled with HKL2000.[13] Structures were solved by molecular replacement with Phaser[14] from the CCP4 program suite based on the coordinates extracted from the structure of MDM2-PMI complex (PDB code: 3EQS) and MDMX-PMI complex (PDB code: 3EQY).[3] The models were refined using Refmac and the structure manually rebuilt with COOT.[15] The MDM2-PMI(8,12)-a complex was refined to R_{factor} of 0.197 and R_{free} of 0.245. The MDMX-PMI(4,8)-a complex was refined to R_{factor} of 0.278 and R_{free} of 0.336. 97.8% and 98.8% of residues fell within allowed regions of the Ramachandran plot as determined by MolProbity, respectively (**Table S3**).

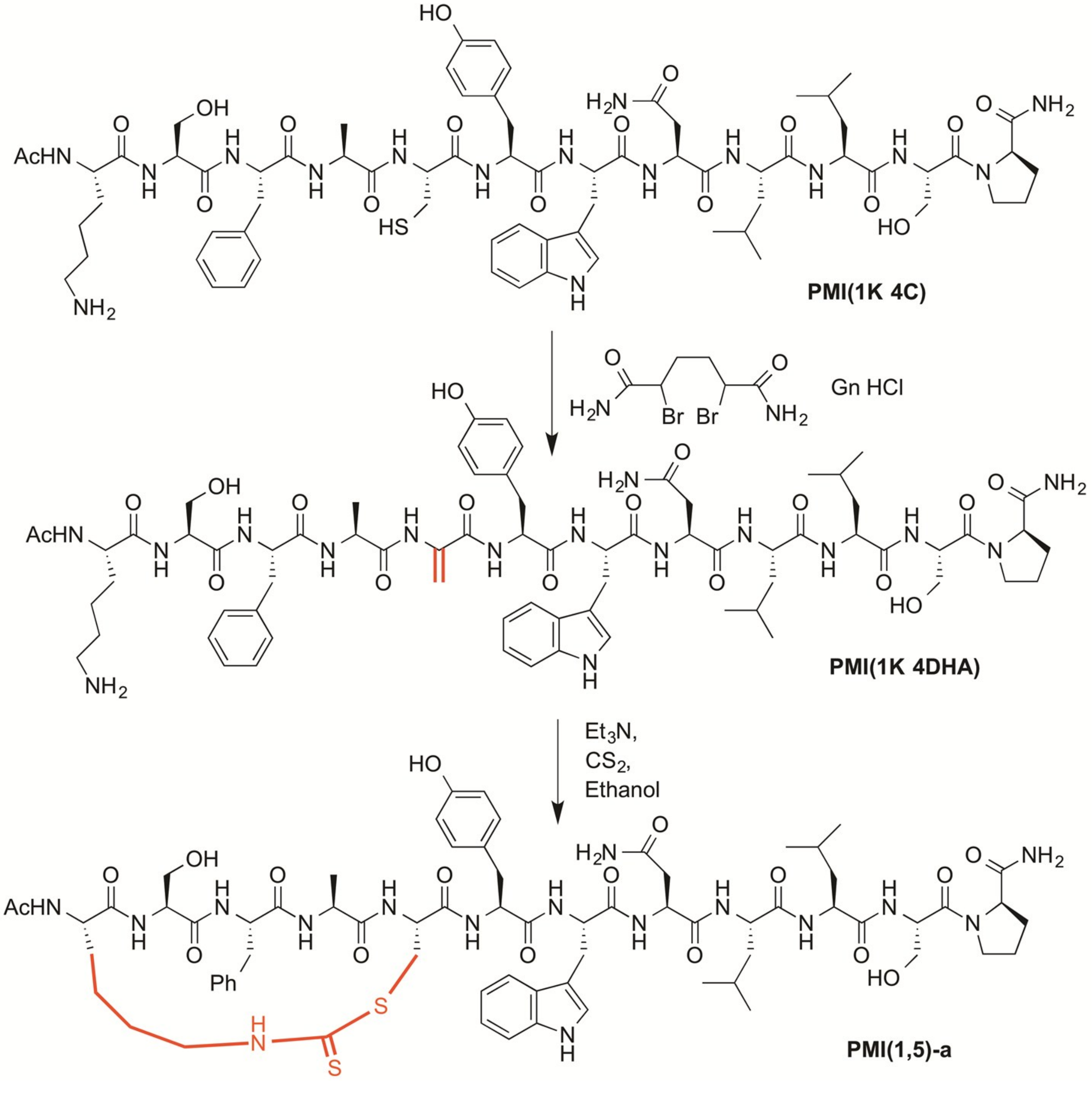

Fig. S1 The synthetic route of DTC-stapled PMI(1,5)-a

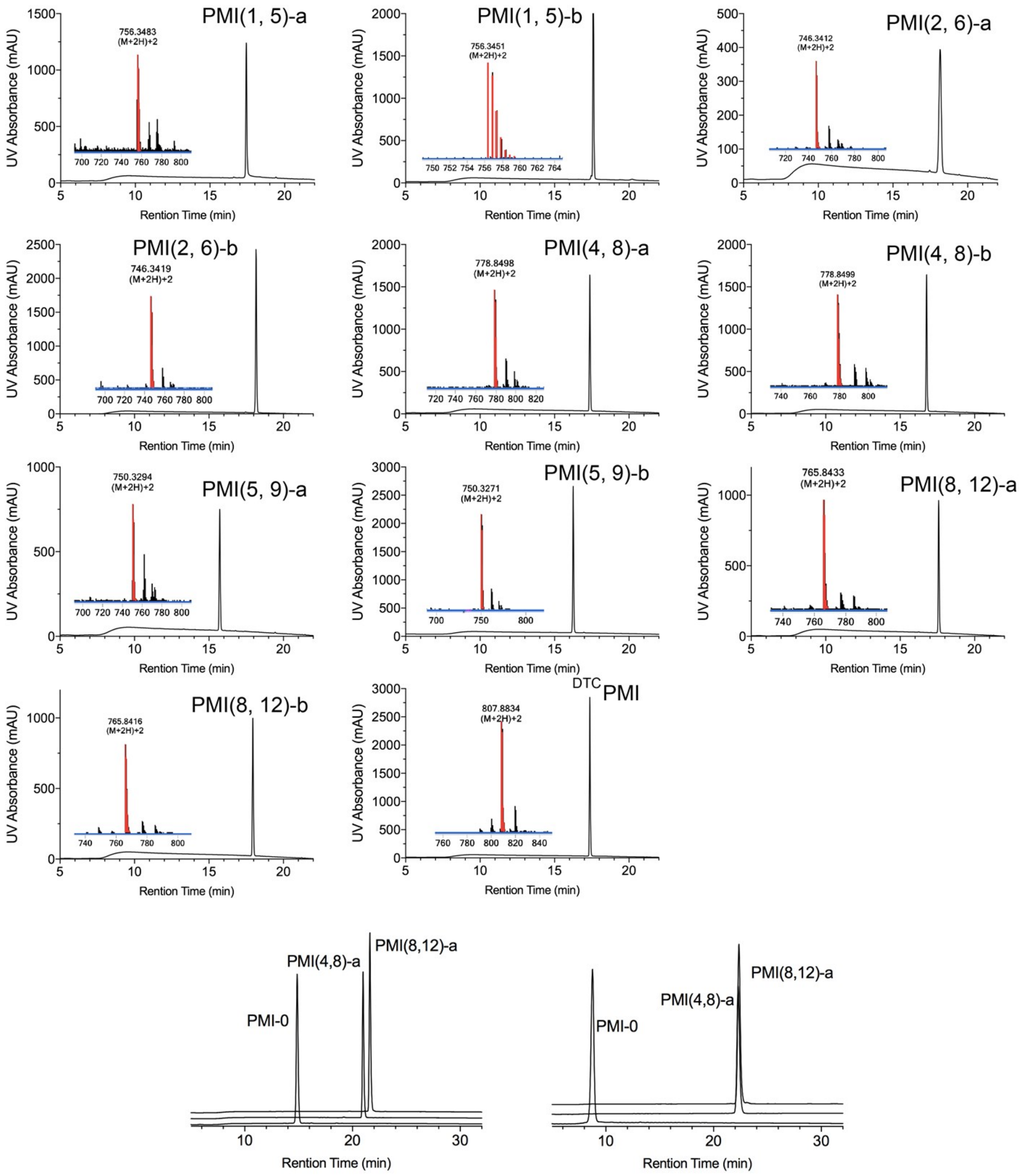

Fig. S2 HPLC chromatograms and MS spectra of DTC-stapled peptides. Bottom panel: PMI-0, PMI(4,8)-a and PMI(8,12)-a analyzed by HPLC at different gradients, 30-60% B (left) and 35-45% B (right) over 30 min (B = acetonitrile).

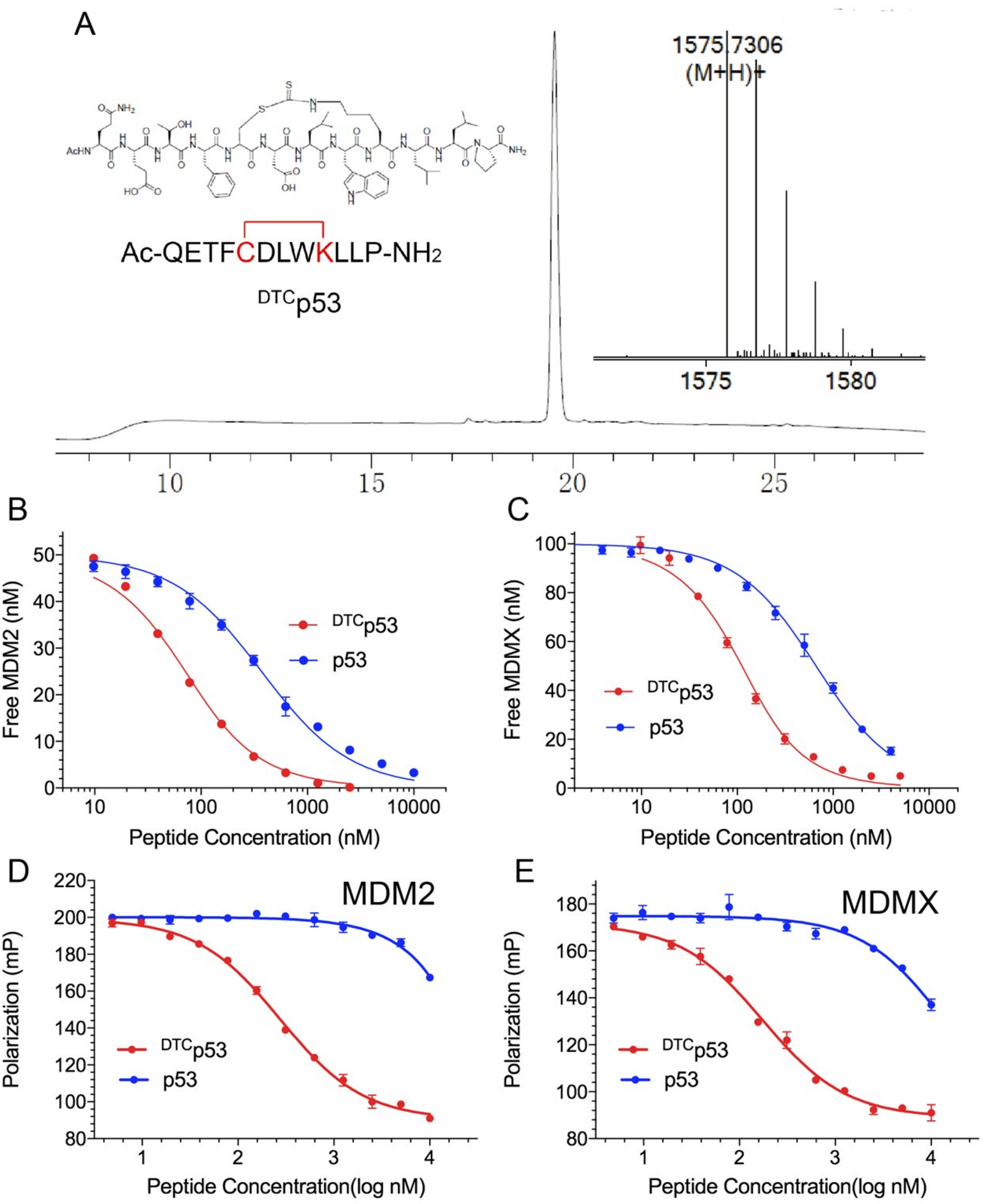

Fig. S3 HPLC and MS chromatograms of ^{DTC}p53 and corresponding binding curves with MDM2 (left) and MDMX (right) as determined by SPR and FP.

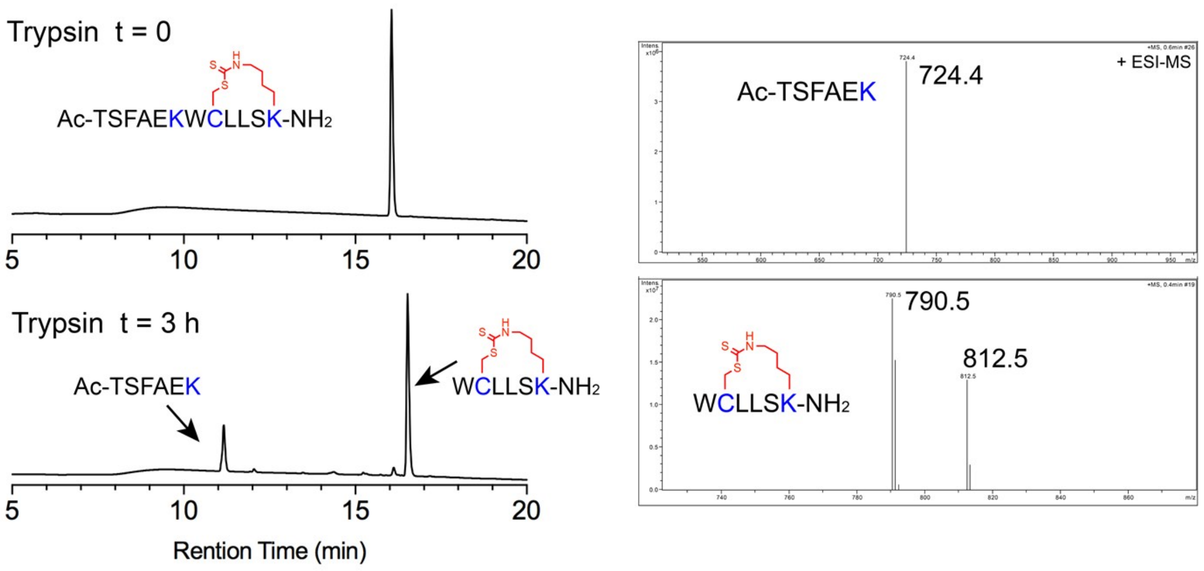

Fig. S4 Tryptic digestion coupled with mass spectrometry analysis confirms the DTC staple formed by Cys and Lys at (*i, i+4*) positions. Note: the 812.5 Da mass peak is the sodium adduct of the DTC-stapled peptide fragment (790.5 Da).

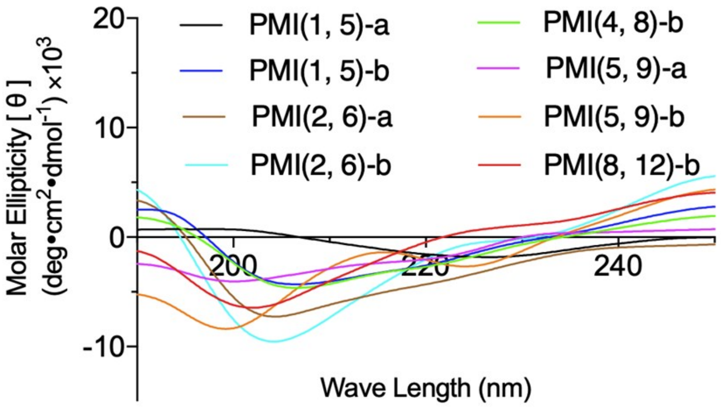

Fig. S5 Circular dichroism spectra of DTC-stapled PMIs

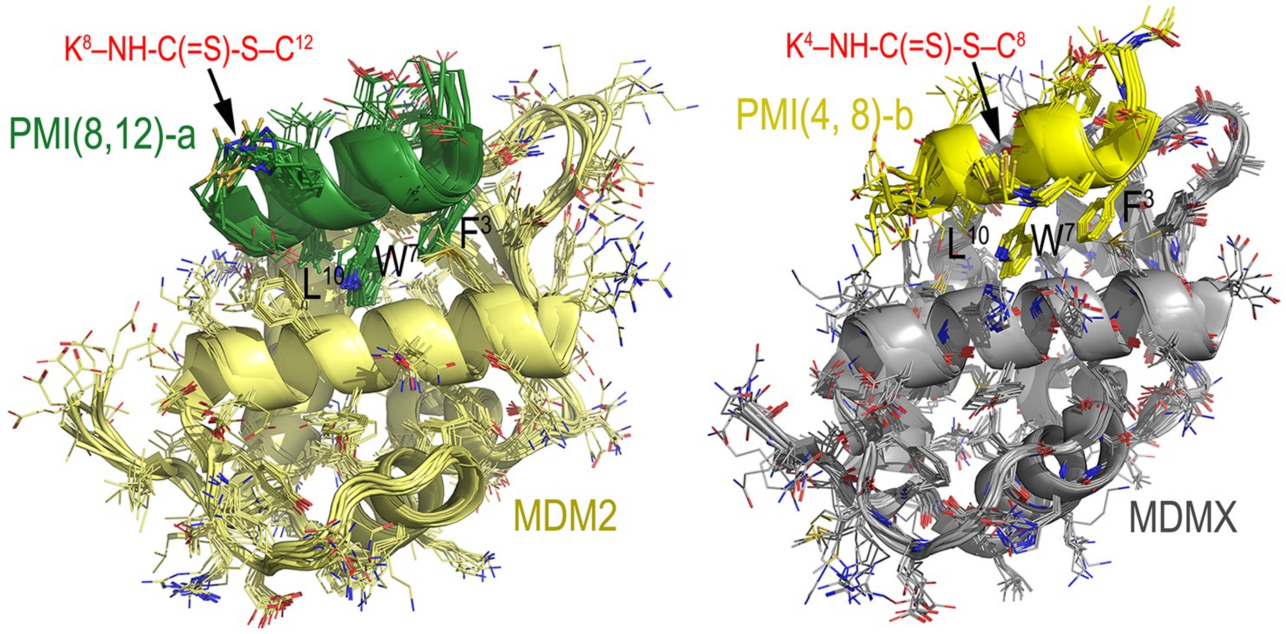

Fig. S6 Superposition of MDM2-PMI(8,12)-a and MDMX-PMI(4,8)-a copies within the asymmetric unit of each crystal form. The root mean square deviation (RMSD) between the 12 copies of MDM2-PMI(8,12)-a complex (left) in the crystal ranges from 0.479 – 1.348 Å, 0.393 – 1.180 Å for MDM2 alone and 0.286 – 1.476 Å for the peptides (**Table S2**). The RMSD between the 8 MDMX-PMI(4, 8)-1 complexes (right) ranges from 0.498 – 0.976 Å in the crystal, 0.368 – 0.774 Å for MDMX and 0.274 – 2.188 Å for PMI(4, 8)-1 (**Table S3**).

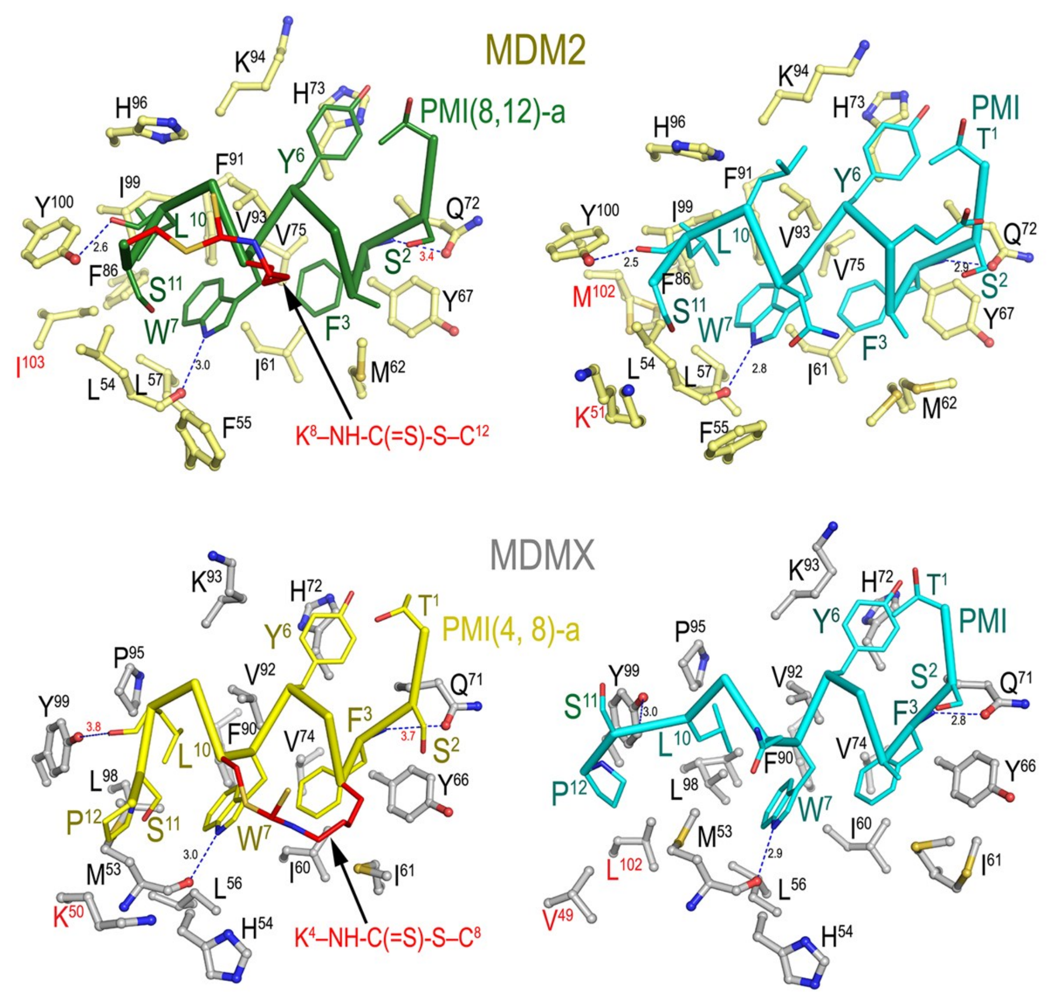

Fig. S7 MDM2-PMI(8,12)-a and MDMX-PMI(4,8)-a complex interfaces. The MDM2-PMI(8,12)-a, MDM2-PMI (PDB code: 3EQS), MDMX-PMI(4,8)-a and MDMX-PMI (PDB code: 3EQY) complex structures were superimposed based on MDM2 (top) and MDMX (bottom). The PMI peptides are shown as ribbon-ball-stick representations. For clarity only side chains of residues of MDM2 and MDMX forming the interface involved in hydrogen bonds and hydrophobic contacts are shown as ball-sticks and residues which differ between the stapled PMI and PMI complexes are colored in red. The same set of residues with the exception of K^{51} and Met^{102} that lines the PMI binding pocket within the MDM2 molecule is involved in PMI(8,12)-a peptide binding (residues 54-55, 57-58, 61-62, 67, 72-73, 75, 86, 91, 93-94, 96, 99-100 of MDM2). In addition, PMI(8,12)-a makes one new hydrophobic contact to I^{103} of MDM2. There are also three direct protein-peptide H-bounds formed at the MDM2-PMI(8,12)-a contact interface (Q^{72} Oε1 to F^{3} N, L^{54} O to W^{3} Nε1, Y^{100} (OH) to L^{10} O) with elongated H-bond of Q^{72} Oε^{1} to F^{3} N. Residues 53-54, 56-57, 60-61, 66, 71-72, 74, 90, 92-93, 95, 98-99 of MDMX line the PMI(4,8)-a binding pocket. The PMI(4,8)-a binding doesn't involve V^{49} and L^{102} of MDMX which are engaged in PMI binding. A new contact to K^{50} of MDMX is formed to accommodate M^{11} of PMI(4,8)-a. There are also two direct protein-peptide H-bounds formed at the MDMX-PMI(4,8)-a contact interface (Q71 Oε1 to F3 N, M^{53} O to W^{3} Nε1 and Y^{99} (OH) to S^{11} O) with elongated H-bond between Q71 Oε1 to F3 N and Y99 (OH) to S11 O).

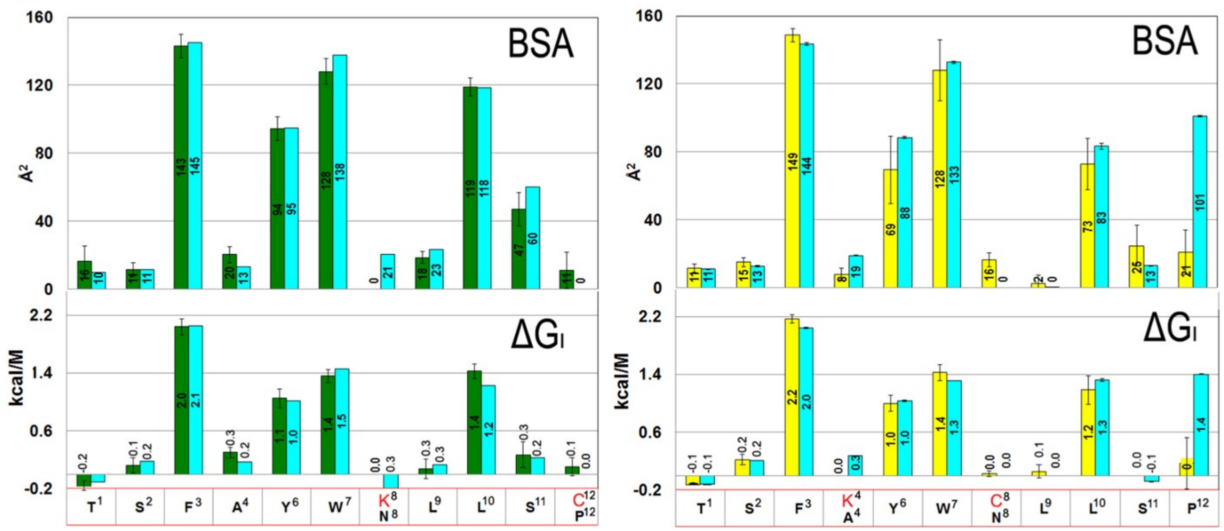

Fig. S8 Structural analysis of interactions of stapled PMI with MDM2 and MDMX. Analysis of the peptide binding interface. The relative contribution of each residue of PMI(8,12)-a and PMI(4,8)-a (green/yellow) and PMI (cyan) to MDM2/MDMX interface is shown as the buried surface area (BSA, top panel) and the solvation energy in kcal/mol (ΔiG, bottom panel) of each position as calculated by PISA. BSA represents the solvent-accessible surface area of the corresponding residue that is buried upon interface formation and the solvation energy gain of the interface is calculated as the difference in solvation energy of a residue between the dissociated and associated structures. A positive solvation energy corresponds to a negative contribution to the solvation energy gain of the interface or put another way, the hydrophobic effect. Hydrogen bonds and salt bridges are not included in ΔiG. When more than one copy of the peptide is present in the asymmetric unit values are shown as the mean with the range displayed as an error bar. The sequence for each position is shown on the bottom. E^5 of PMI peptides is not shown since it is not contributing to the binding in any of complex shown.

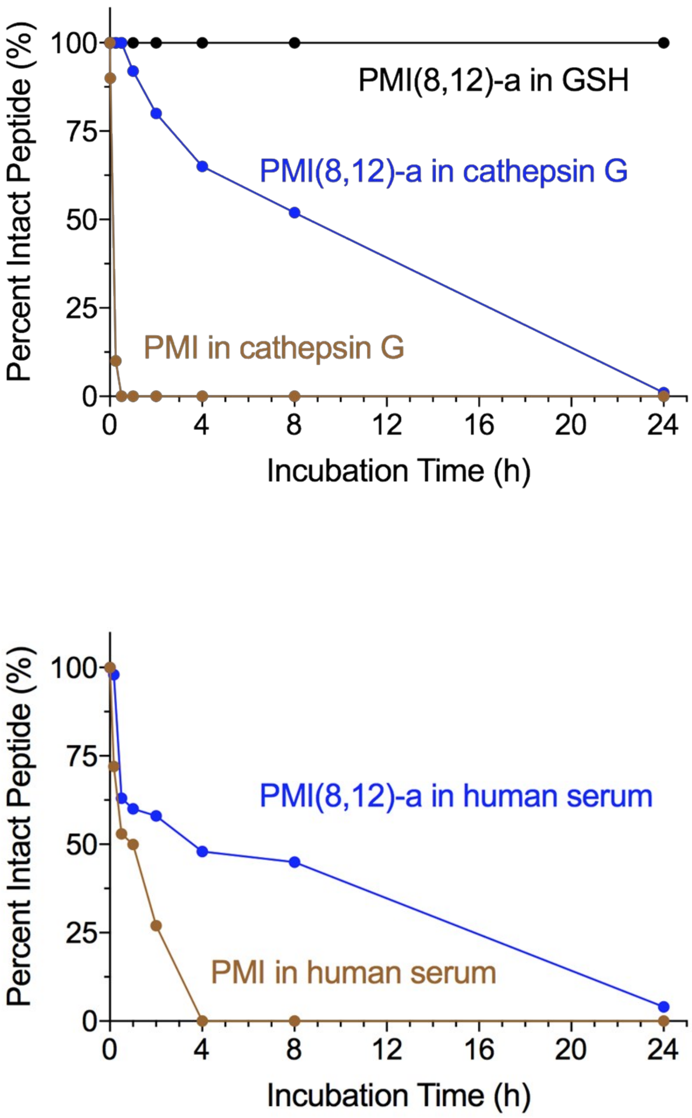

Fig. S9 Top panel: Stability of PMI-0 and PMI(8,12)-a in the presence of cathepsin G or GSH as monitored by HPLC. **Bottom panel:** Stability of PMI-0 and PMI(8,12)-a in the presence of human serum.

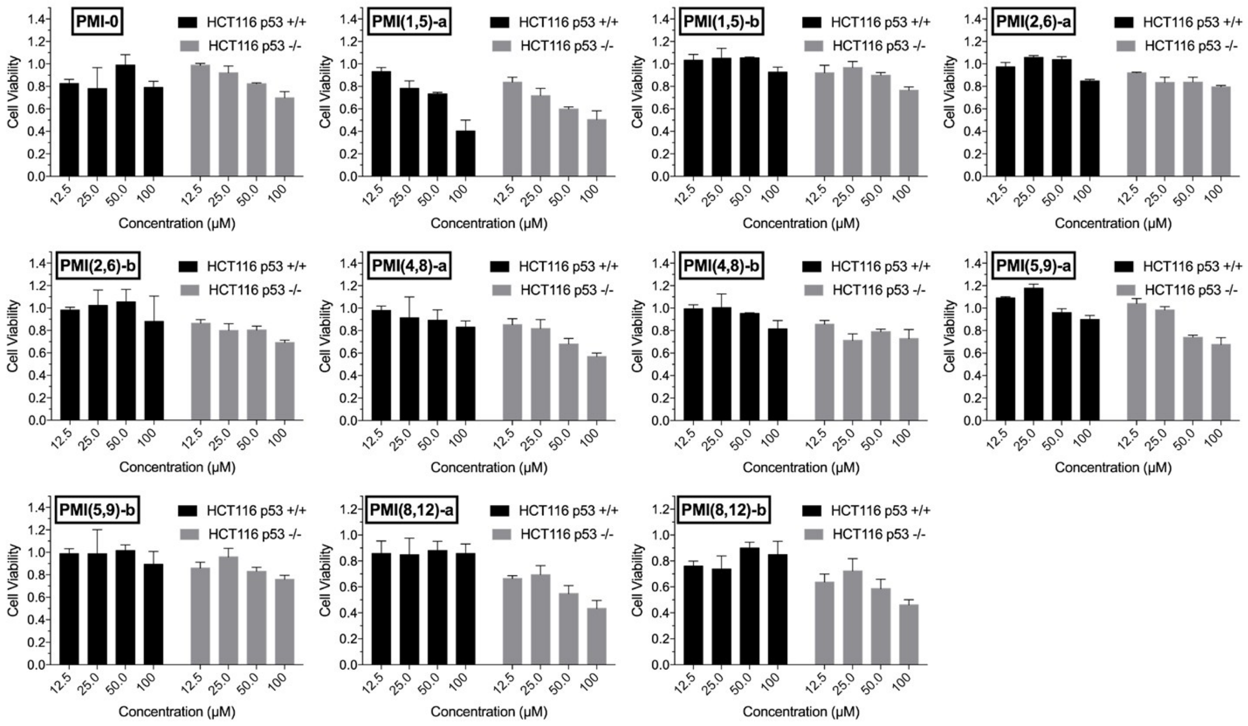

Fig. S10 Viability of HCT116 p53$^{+/+}$ and HCT116 p53$^{-/-}$ cell lines in the presence of PMI-0 and stapled PMI peptides.

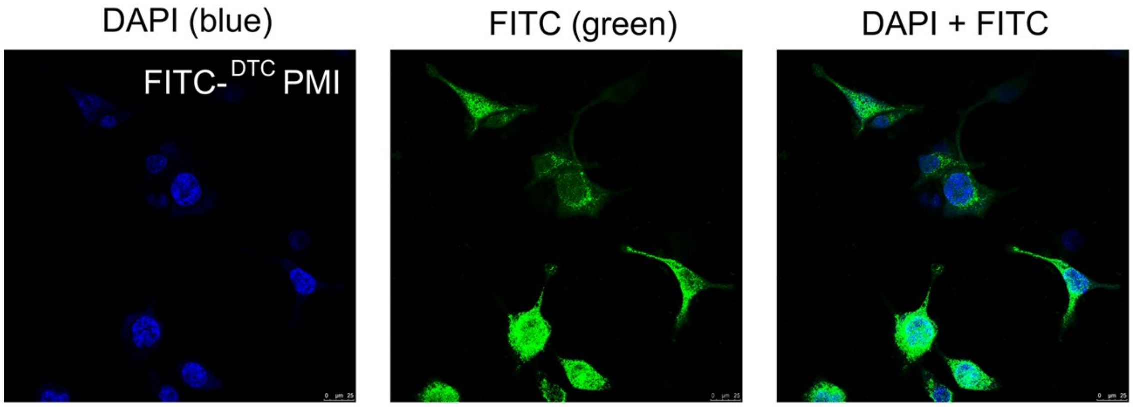

Fig. S11 Confocal microscope images of FITC-labeled ^{DTC}PMI localization in HCT116 cells.

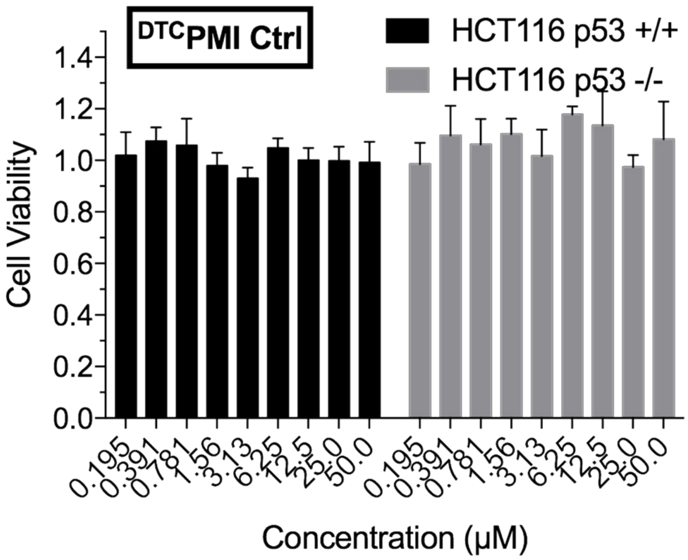

Fig. S12 Viability of HCT116 p53$^{+/+}$ and HCT116 p53$^{-/-}$ cell lines in the presence of linear ^{DTC}PMI control.

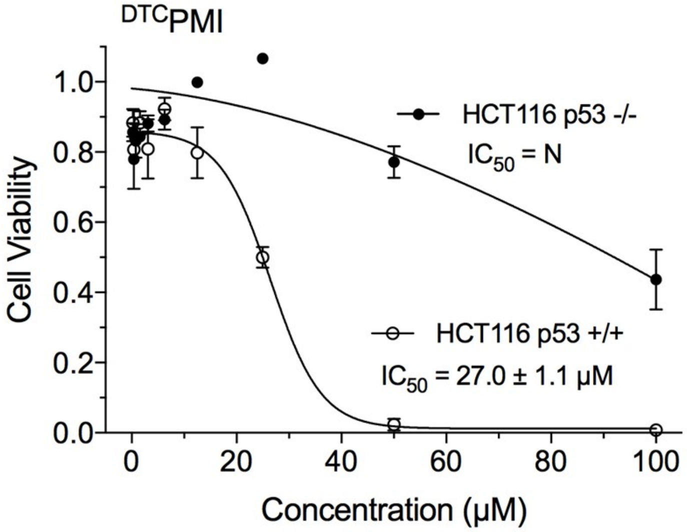

Fig. S13 Fitted curves of the viability of HCT116 *p53*$^{+/+}$ and HCT116 p53$^{-/-}$ cell lines in the presence of ^{DTC}PMI.

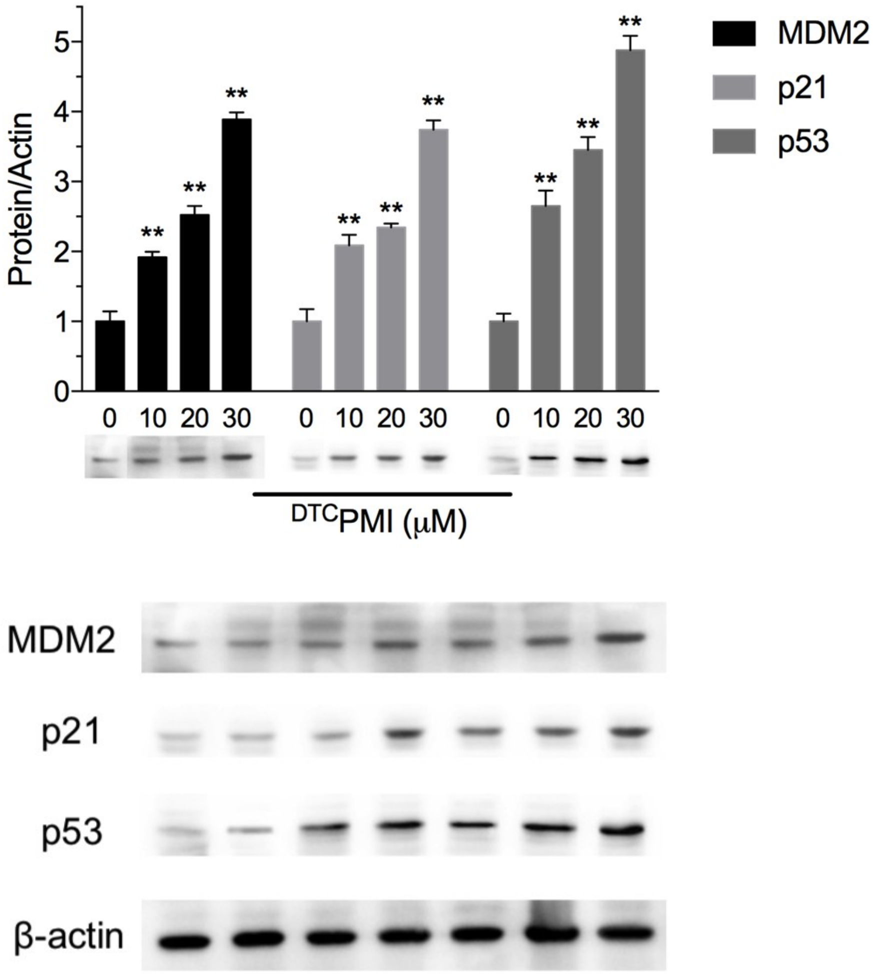

Fig. S14 Top panel: Quantitative Western blot analysis of MDM2, p21 and p53 in HCT116 *p53*$^{+/+}$ cells treated with different concentrations of ^{DTC}PMI. **Bottom panel:** Original western blot gel for MDM2, p21 and p53. Lane 1 was for blank control and lanes 5-7 were for ^{DTC}PMI. Three additional lanes (lane 2-4) for peptide samples were unrelated to this work.

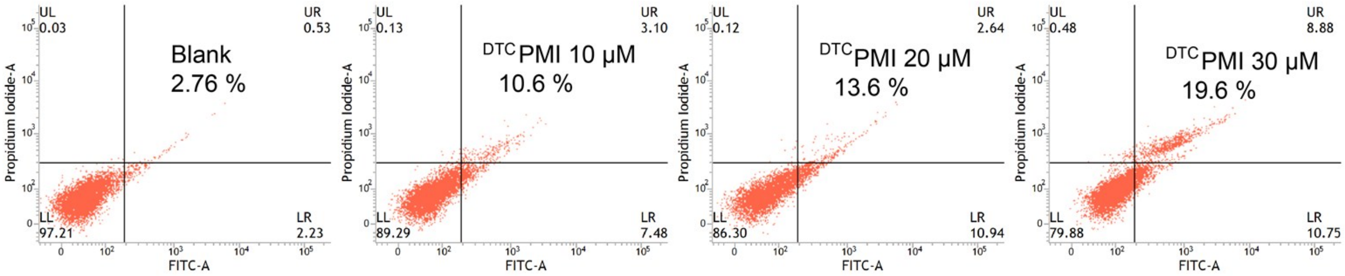

Fig. S15 ^{DTC}PMI-induced apoptosis of HCT 116 p53$^{+/+}$ cells as measured by flow cytometry.

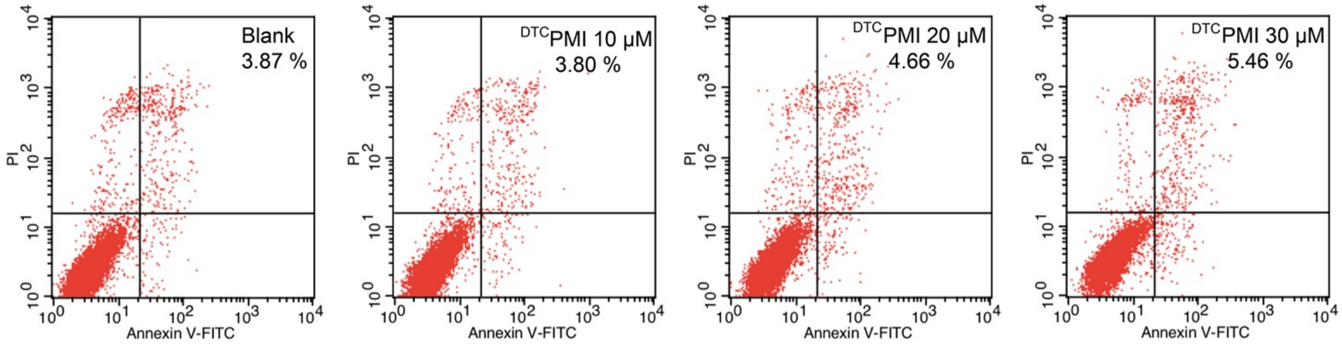

Fig. S16 ^{DTC}PMI-induced apoptosis of HCT 116 p53$^{-/-}$ cells as measured by flow cytometry.

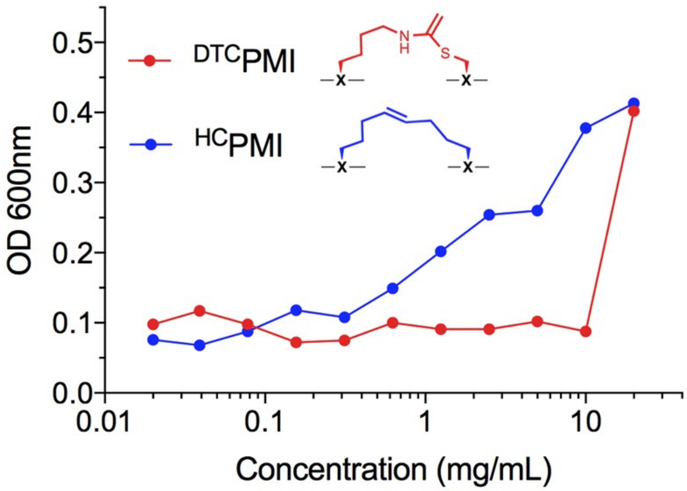

Fig. S17 Comparison of solubility between DTC- and Hydrocarbon-stapled PMI.

Table S1 Yield and HR-MS spectrometry data for DTC-stapled PMI peptides.

Compound	Yield (%)	Calculated Mass (M/2+H)	Found	Method
PMI(1,5)-a	28	756.3441	756.3483	Q-TOF
PMI(1,5)-b	37	756.3441	756.3451	Q-TOF
PMI(2, 6)-a	34	746.3416	746.3412	Q-TOF
PMI(2, 6)-b	39	746.3416	746.3419	Q-TOF
PMI(4, 8)-a	42	778.8493	778.8498	Q-TOF
PMI(4, 8)-b	33	778.8493	778.8499	Q-TOF
PMI(5, 9)-a	24	750.3259	750.3294	Q-TOF
PMI(5, 9)-b	27	750.3259	750.3271	Q-TOF
PMI(8, 12)-a	45	765.8414	765.8433	Q-TOF
PMI(8, 12)-b	38	765.8414	765.8416	Q-TOF
^{DTC}PMI	46	807.8814	807.8834	Q-TOF

Table S2 Data collection and refinement statistics

Data collection	MDM2-PMI(8,12)-a	MDMX-PMI(4,8)-a
Wavelength, Å	0.97946	0.97946
Space group	$C222_1$	P1
Cell parameters		
a, b, c, Å	90.8, 157.5, 196.7	43.3, 47.7, 93.4
α, β, γ, °	90.0, 90.0, 90.0	76.7, 89.9, 72.6
Complexes/a.u.	12	8
Resolution, (Å)	50-1.8 (1.83-1.8)	50-2.7 (2.75-2.7)
# of reflections		
Total	419,465	31,027
Unique	123,372	16,330
$R_{merg}{}^{b}$, %	7.6 (29.9)	13.2 (61.4)
I/σ	23.2 (3.0)	10.3 (1.1)
Completeness, %	95.5 (97.9)	86.0 (78.7)
Redundancy	3.4 (3.4)	1.9 (1.8)
**Refinement Statistics**		
Resolution, Å	41.7 - 1.80	50 – 2.7
R^c, %	19.8	28.1
$R_{free}{}^{d}$, %	24.6	33.5
# of atoms		
Protein	9,547	5,910
Water	418	27
Ligand/Ion	182	115
Overall B value $(Å)^2$		
Protein	26.8	54.4
Water	24.8	38.9
Ligand/Ion	26.7	57.7
Root mean square deviation		
Bond lengths, Å	0.012	0.006
Bond angles, °	1.79	1.14
Ramachandran [e]		
favored, %	91.2	95.5
allowed, %	6.6	3.3
outliers, %	2.2	1.2
PDB ID	5VK0	5VK1

[a]all data (outer shell).
[b]$R_{merge} = \sum | I - \langle I \rangle | / \sum I$, where I is the observed intensity and $\langle I \rangle$ is the average intensity obtained from multiple observations of symmetry-related reflections after rejections
[c]$R = \sum \| F_o | - | F_c \| / \sum | F_o |$, where F_o and F_c are the observed and calculated structure factors, respectively
[d]R_{free} = as defined by Brünger

Table S3 The root mean square deviation (RMSD) between MDM2-PMI(8,12)-a and MDM2-PMI complexes. Comparisons were made between 12 copies of MDM2-PMI(8,12) complex (copies a, b, c, d, e, f, g, h, i, j, k, l) and one copy of MDM2-PMI complex.

	MDM2-PMI(8,12)a	MDM2-PMI(8,12)b	MDM2-PMI(8, 12)c	MDM2-PMI(8, 12)d	MDM2-PMI(8, 12)e	MDM2-PMI(8, 12)f	MDM2-PMI(8, 12)g	MDM2-PMI(8, 12)h	MDM2-PMI(8, 12)i	MDM2-PMI(8, 12)j	MDM2-PMI(8, 12)k	MDM2-PMI(8, 12)l	MDM2-PMI
MDM2-PMI(8,12)a	-	0.686	0.927	0.647	0.579	0.835	0.526	0.926	0.702	1.038	1.120	0.722	0.914
PMI(8,12)a	-	0.759	0.867	0.751	0.589	0.871	0.384	1.017	0.632	0.833	0.952	0.853	0.731
MDM2-1	-	0.583	0.868	0.567	0.510	0.772	0.468	0.870	0.662	0.994	1.044	0.650	0.885
MDM2-PMI(8,12)b	0.686	-	1.049	0.620	0.548	0.686	0.574	0.873	0.779	1.064	1.045	0.752	0.754
PMI(8,12)b	0.759	-	1.240	1.225	0.942	0.580	0.670	0.756	1.043	1.262	1.004	1.275	0.678
MDM2b	0.583	-	0.818	0.393	0.435	0.676	0.543	0.884	0.666	0.823	1.015	0.578	0.745
		-											
MDM2-PMI(8,12)c	0.927	1.049	-	0.917	1.037	1.133	1.072	1.090	0.802	0.902	1.348	0.810	0.927
PMI(8,12)c	0.867	1.240	-	0.645	1.114	1.374	1.043	1.340	0.753	0.647	1.276	0.661	0.897
MDM2c	0.868	0.818	-	0.825	0.860	0.915	0.935	0.898	0.699	0.877	1.180	0.713	0.851
MDM2-PMI(8,12)d	0.647	0.620	0.917	-	0.538	0.826	0.603	0.959	0.569	1.007	1.087	0.520	0.783
PMI(8,12)d	0.751	1.225	0.645	-	0.850	1.288	0.880	1.394	0.286	0.573	1.258	0.319	0.832
MDM2d	0.567	0.393	0.825	-	0.426	0.648	0.533	0.845	0.594	0.900	0.988	0.539	0.760
MDM2-PMI(8,12)e	0.579	0.548	1.037	0.538	-	0.721	0.520	0.927	0.696	1.101	1.061	0.703	0.786
PMI(8,12)e	0.589	0.942	1.114	0.850	-	0.852	0.729	1.172	0.662	0.890	1.158	0.989	0.643
MDM2e	0.510	0.435	0.860	0.426	-	0.669	0.456	0.875	0.663	0.947	0.973	0.603	0.782

MDM2-PMI(8,12)f	0.835	0.686	1.133	0.826	0.721	-	0.760	0.890	0.852	1.186	1.028	0.866	0.770
PMI(8,12)f	0.871	0.580	1.374	1.288	0.852	-	0.786	0.776	1.088	1.319	1.056	1.385	0.490
MDM2f	0.772	0.676	0.915	0.648	0.669	-	0.720	0.884	0.732	1.017	0.976	0.671	0.774
MDM2-PMI(8,12)g	0.526	0.574	1.072	0.603	0.520	0.760	-	0.960	0.783	1.144	1.062	0.748	0.896
PMI(8,12)g	0.384	0.670	1.043	0.880	0.729	0.786	-	0.937	0.817	1.030	0.884	0.974	0.620
MDM2g	0.468	0.543	0.935	0.533	0.456	0.720	-	0.953	0.748	1.011	1.042	0.688	0.912
MDM2-PMI(8,12)h	0.926	0.873	1.090	0.959	0.927	0.890	0.960	-	0.791	1.055	0.956	0.877	0.818
PMI(8,12)h	1.017	0.756	1.340	1.394	1.172	0.776	0.937	-	1.315	1.417	1.134	1.476	0.768
MDM2h	0.870	0.884	0.898	0.845	0.875	0.884	0.953	-	0.638	0.784	0.860	0.710	0.799
MDM2-PMI(8,12)i	0.702	0.779	0.802	0.569	0.696	0.852	0.783	0.791	-	0.785	1.139	0.479	0.607
PMI(8,12)i	0.632	1.043	0.753	0.286	0.662	1.088	0.817	1.315	-	0.501	1.337	0.314	0.560
MDM2i	0.662	0.666	0.699	0.594	0.663	0.732	0.748	0.638	-	0.600	1.013	0.494	0.594
MDM2-PMI(8,12)j	1.038	1.064	0.902	1.007	1.101	1.186	1.144	1.055	0.785	-	1.299	0.850	0.878
PMI(8,12)j	0.833	1.262	0.647	0.573	0.890	1.319	1.030	1.417	0.501	-	1.373	0.547	0.859
MDM2j	0.994	0.823	0.877	0.900	0.947	1.017	1.011	0.784	0.600	-	1.110	0.704	0.714
MDM2-PMI(8,12)k	1.120	1.045	1.348	1.087	1.061	1.028	1.062	0.956	1.139	1.299	-	1.045	0.970
PMI(8,12)k	0.952	1.004	1.276	1.258	1.158	1.056	0.884	1.134	1.337	1.373	-	1.340	0.829
MDM2k	1.044	1.015	1.180	0.988	0.973	0.976	1.042	0.860	1.013	1.110	-	0.923	0.984

MDM2-PMI(8,12)l	0.722	0.752	0.810	0.520	0.703	0.866	0.748	0.877	0.479	0.850	1.045	-	0.606
PMI(8,12)l	0.853	1.275	0.661	0.319	0.989	1.385	0.974	1.476	0.314	0.547	1.340	-	0.854
MDM2l	0.650	0.578	0.713	0.539	0.603	0.671	0.688	0.710	0.494	0.704	0.923	-	0.542
MDM2-PMI	0.914	0.754	0.927	0.783	0.786	0.770	0.896	0.818	0.607	0.878	0.970	0.606	-
PMI	0.731	0.678	0.897	0.832	0.643	0.490	0.620	0.768	0.560	0.859	0.829	0.854	-
MDM2	0.885	0.745	0.851	0.760	0.782	0.774	0.912	0.799	0.594	0.714	0.984	0.542	-

Table S4 The root mean square deviation (RMSD) between MDMX-PMI(4,8)-a and MDMX-PMI complexes. Comparisons were made between 8 copies of MDMX-PMI(4, 8) complex (copies a, b, c, d, e, f, g, h) and two copies of MDM-PMI complex (copies a, b).

	MDMX-PMI(4, 8)a	MDMX-PMI(4, 8)b	MDMX-PMI(4, 8)c	MDMX-PMI(4, 8)d	MDMX-PMI(4, 8)e	MDMX-PMI(4, 8)f	MDMX-PMI(4, 8)g	MDMX-PMI(4, 8)h	MDMX-PMIa	MDMX-PMIb
MDMX-PMI(4, 8)a	-	0.976	0.600	0.677	0.936	0.762	0.586	0.589	1.254	1.270
PMI(4, 8)a	-	2.188	1.011	1.091	1.815	1.100	0.721	0.274	1.059	1.081
MDMXa	-	0.464	0.479	0.538	0.461	0.641	0.529	0.594	0.966	0.969
MDMX-PMI(4, 8)b	0.976	-	0.618	0.545	0.660	0.830	0.724	0.611	1.534	1.574
PMI(4, 8)b	2.188	-	1.018	0.752	1.089	1.002	1.411	0.590	2.461	2.582
MDMXb	0.464	-	0.505	0.454	0.504	0.735	0.526	0.583	0.853	0.855
		-								
MDMX-PMI(4, 8)c	0.600	0.618	-	0.684	0.498	0.761	0.599	0.672	1.096	1.096
PMI(4, 8)c	1.011	1.018	-	1.029	0.946	1.010	1.003	1.060	0.902	0.902
MDMXc	0.479	0.505	-	0.575	0.368	0.683	0.491	0.575	0.939	0.940
MDMX-PMI(4, 8)d	0.677	0.545	0.684	-	0.543	0.756	0.647	0.627	1.106	1.107
PMI(4, 8)d	1.091	0.752	1.029	-	0.732	0.464	1.157	0.354	1.318	1.319
MDMXd	0.538	0.454	0.575	-	0.490	0.744	0.471	0.632	0.845	0.847
MDMX-PMI(4, 8)e	0.936	0.660	0.498	0.543	-	0.733	0.711	0.564	1.508	1.541
PMI(4, 8)e	1.815	1.089	0.946	0.732	-	1.036	1.338	0.585	2.112	2.198
MDMXe	0.461	0.504	0.368	0.490	-	0.617	0.463	0.552	0.896	0.899

MDMX-PMI(4, 8)f	0.762	0.830	0.761	0.756	0.733	-	0.816	0.746	1.388	1.389
PMI(4, 8)f	1.100	1.002	1.010	0.464	1.036	-	0.945	0.319	1.104	1.105
MDMXf	0.641	0.735	0.683	0.744	0.617	-	0.710	0.774	1.113	1.114
MDMX-PMI(4, 8)g	0.586	0.724	0.599	0.647	0.711	0.816	-	0.501	1.036	1.037
PMI(4, 8)g	0.721	1.411	1.003	1.157	1.338	0.945	-	0.366	0.453	0.456
MDMXg	0.529	0.526	0.491	0.471	0.463	0.710	-	0.496	0.914	0.916
MDMX-PMI(4, 8)h	0.589	0.611	0.672	0.627	0.564	0.746	0.501	-	1.066	1.067
PMI(4, 8)h	0.274	0.590	1.060	0.354	0.585	0.319	0.366	-	0.362	0.364
MDMXh	0.594	0.583	0.575	0.632	0.552	0.774	0.496	-	0.955	0.956
MDMX-PMIa	1.254	1.534	1.096	1.106	1.508	1.388	1.036	1.066	-	
PMIa	1.059	2.461	0.902	1.318	2.112	1.104	0.453	0.362	-	0.107
MDMXa	0.966	0.853	0.939	0.845	0.896	1.113	0.914	0.955	-	0.289
										0.017
MDMX-PMIb	1.270	1.574	1.096	1.107	1.541	1.389	1.037	1.067	0.107	-
PMIb	1.081	2.582	0.902	1.319	2.198	1.105	0.456	0.364	0.289	-
MDMXb	0.969	0.855	0.940	0.847	0.899	1.114	0.916	0.956	0.017	-

References

1 P. E. Dawson, S. B. Kent, *Annu. Rev. Biochem,* 2000, **69**, 923-960.

2 P. E. Dawson, T. W. Muir, I. Clark-Lewis, S. B. Kent, *Science,* 1994, **266**, 776-779.

3 M. Pazgier, M. Liu, G. Zou, W. Yuan, C. Li, C. Li, J. Li, J. Monbo, D. Zella, S. G. Tarasov, W. Lu, *Proc. Natl. Acad. Sci. U. S. A.,* 2009, **106**, 4665-4670.

4 S. B. Kent, *Chem. Soc. Rev.,* 2009, **38**, 338-351.

5 X. Chen, L. Tai, J. Gao, J. Qian, M. Zhang, B. Li, C. Xie, L. Lu, W. Lu, W. Lu, *J. Control. Release,* 2015, **218**, 29-35.

6 C. Li, M. Pazgier, C. Li, W. Yuan, M. Liu, G. Wei, W. Y. Lu, W. Lu, *J. Mol. Biol.,* 2010, **398**, 200-213.

7 M. Liu, C. Li, M. Pazgier, C. Li, Y. Mao, Y. Lv, B. Gu, G. Wei, W. Yuan, C. Zhan, W. Y. Lu, W. Lu, *Proc. Natl. Acad. Sci. U. S. A.,* 2010, **107**, 14321-14326.

8 M. Liu, M. Pazgier, C. Li, W. Yuan, C. Li, W. Lu, *Angew. Chem. Int. Ed. Engl.,* 2010, **49**, 3649-3652.

9 C. Zhan, K. Varney, W. Yuan, L. Zhao, W. Lu, *J. Am. Chem. Soc.,* 2012, **134**, 6855-6864.

10 C. Zhan, L. Zhao, X. Wei, X. Wu, X. Chen, W. Yuan, W. Y. Lu, M. Pazgier, W. Lu, *J. Med. Chem.,* 2012, **55**, 6237-6241.

11 K. Ding, Y. Lu, Z. Nikolovska-Coleska, S. Qiu, Y. Ding, W. Gao, J. Stuckey, K. Krajewski, P. P. Roller, Y. Tomita, D. A. Parrish, J. R. Deschamps, S. Wang, *J. Am. Chem. Soc.,* 2005, **127**, 10130-10131.

12 D. Wang, K. Chen, J. L. Kulp Iii, P. S. Arora, *J. Am. Chem. Soc.,* 2006, **128**, 9248-9256.

13 Z. Otwinowski, W. Minor, Charles W. Carter, Jr., in *Methods in Enzymology, Vol. Volume 276*, Academic Press, **1997**, pp. 307-326.

14 G. N. Murshudov, A. A. Vagin, E. J. Dodson, *Acta Crystallogr D Biol Crystallogr,* 1997, **53**, 240-255.

15 P. Emsley, K. Cowtan, *Acta Crystallogr D Biol Crystallogr,* 2004, **60**, 2126-2132.
